# Genomic and functional co-diversification imprint African Hominidae microbiomes to signal dietary and lifestyle adaptations

**DOI:** 10.1080/19490976.2025.2484385

**Published:** 2025-03-31

**Authors:** Saria Otani, Marie Louise Jespersen, Christian Brinch, Frederik Duus Møller, Bo Pilgaard, Emilie Egholm Bruun Jensen, Pimlapas Leekitcharoenphon, Christina Aaby Svendsen, Amalie H. Aarestrup, Tolbert Sonda, Teresa J. Sylvina, Jeff Leach, Alexander Piel, Fiona Stewart, Panagiotis Sapountzis, Paul E. Kazyoba, Happiness Kumburu, Frank M. Aarestrup

**Affiliations:** a Research group for Genomic Epidemiology, National Food Institute, Technical University of Denmark, Lyngby, Denmark; b Novo Nordisk Foundation Center for Protein Research, Faculty of Health and Medical Sciences, University of Copenhagen, Copenhagen, Denmark; c Department of Biotechnology and Biomedicine, Section for Protein Chemistry and Enzyme Technology, Technical University of Denmark, Lyngby, Denmark; d Biotechnology Research Laboratory, Kilimanjaro Clinical Research Institute (KCRI), Moshi, Tanzania; e Kilimanjaro Christian Medical Centre (KCMC), Moshi, Tanzania; f Department of Microbiology, Kilimanjaro Christian Medical University College (KCMUCo), Moshi, Tanzania; g National Academies of Sciences, Engineering and Medicine, Washington, DC, USA; h Department of Veterinary and Biomedical Sciences, The Pennsylvania State University, State College, PA, USA; i Microbiome Network and Department of Agricultural Biology, Colorado State University, Fort Collins, CO, USA; j Department of Human Origins, Max Planck Institute of Evolutionary Anthropology, Leipzig, Germany; k Department of Anthropology, University College London, London, UK; l School of Biological and Environmental Sciences, Liverpool John Moores University, Liverpool, UK; m Université Clermont Auvergne, INRAE, UMR 0454 MEDIS, Clermont-Ferrand, France; n National Institute for Medical Research, Dar-Es-Salaam, Tanzania

**Keywords:** Microbiome, evolution, host adaptation, African hominidae

## Abstract

In the diverse landscape of African hominids, the obligate relationship between the host and its microbiome narrates signals of adaptation and co-evolution. Sequencing 546 African hominid metagenomes, including those from indigenous Hadza and wild chimpanzees, identified similar bacterial richness and diversity surpassing those of westernized populations. While hominids share core bacterial communities, they also harbor distinct, population-specific bacterial taxa tailored to specific diets, ecology and lifestyles, differentiating non-indigenous and indigenous humans and chimpanzees. Even amongst shared bacterial communities, several core bacteria have co-diversified to fulfil unique dietary degradation functions within their host populations. These co-evolutionary trends extend to non-bacterial elements, such as mitochondrial DNA, antimicrobial resistance, and parasites. Our findings indicate that microbiome-host co-adaptations have led to both taxonomic and within taxa functional displacements to meet host physiological demands. The microbiome, in turn, transcends its taxonomic interchangeable role, reflecting the lifestyle, ecology and dietary history of its host.

## Introduction

Gut microbiomes often reflect their host phylogeny, environments, physiology, and lifestyle.^1–6^ The family Hominidae includes humans and chimpanzees, gorillas, and orangutans^7^ which share, besides their evolutionary relationship, relatively similar diets, habitats, and social behaviors.^8^ Comparative studies of Hominidae gut microbiomes both within and between different genera could therefore help to reveal the extent and nature of microbiome adaptation and evolution.^1–6,9,10^

Several microbiome adaptations have been noted in Hominidae primates. For instance, chimpanzees consuming fruit-rich, low-protein diets have microbiomes dominated by bacterial taxa capable of fermenting plant-based carbohydrates,^4^ and there are known geographic differences in human gut microbiomes, likely due to differences in diet, hygiene, and lifestyle.^11–15^ The microbiomes of individuals belonging to different Hominidae genera (*e.g*., *Homo* and *Pan*) are distinct,^1,4^ suggesting that social behavior and ecology may also help shape individual microbiomes.^6,16^ Microbiomes can also help hosts adapt to their (local) physical environments, with, for example, the microbiomes of a number of primate species living in cold environments containing bacteria that produce heat-generating metabolites that could help to maintain body temperature in cold conditions.^17^

The co-evolutionary relationship of Hominidae and their microbiomes is also mirrored by their gene reservoir and enzyme functional adaptations.^1,4^ For example, migration from a non-western nation to the United States has been shown to cause a decrease in gut microbiome diversity,^13^ likely driven by dietary adaptations that alter microbiome enzymatic capacity, such as an increased abundance of carbohydrate-active enzymes (CAZymes) associated with shifts to animal product-rich or plant-based diets.^5,13,18^ Nevertheless, there are significant gaps in our knowledge about the interactions between microbiomes and all exogenous stimuli. For instance, little is known about how hominid microbiomes have adapted to infectious diseases and associated antimicrobial resistance. To our knowledge, there has been only one study of wild ape resistomes (reservoir of antibiotic-resistance genes in a microbial niche) which showed that lifestyle influences microbiome composition, and its resistome, more than geography or species.^19^

Comparative microbiome studies are therefore highly valuable for revealing the extent and nature of adaptive responses. Most existing studies have been biased toward European or North American microbiomes,^5,6,8,11–13^ with few studies conducted in African or South American populations^20,21^ or in indigenous populations.^1,15,22–25^ The gut microbiomes of the Hadza, hunter-gatherers from northern Tanzania with a diet dominated by tubers, baobab, seasonal fruit, honey, and hunted animals,^26^ have attracted particular attention due to their especially rich and diverse gut microbiomes. Hadza gut microbiome composition varies according to season and hence food availability, suggesting high microbiome plasticity.^22,25^ Studies have compared Hadza microbiomes to western microbiomes (United States or Europe),^15,23,24^ neglecting their immediate environment in Africa that might have shaped their microbiome communities. This resulted in limited available information on the microbiome and its diversity among other rural and urban populations in Africa in general, including Hadza homeland and the Batongwe people in Mahale Mountains in Tanzania.

A similar limitation is also evident in chimpanzee gut microbiomes. Where the studies conducted so far have focused mainly on kinship, age, geographical and social influences on the gut microbiome, especially the variations between jungle-living and savanna-living lifestyles,^9,19,27–29^ rather than addressing their symbiotic relationships or co-evolution with gut microbes or with their closest relatives in humans. While attempts have been made to characterize the composition and diversity of microbial communities within Hominidae primates,^1,3,4,10^ few have investigated the rooted symbiotic relationship and fewer still co-speciation (in this context: parallel evolutionary divergence of hosts and their associated microbes) and phylosymbiosis (in this context: microbiome similarity reflecting host phylogenetic relationships) between primate hosts and their microbiomes and consequent functional diet or lifestyle adaptations.^1,10^ The majority of existing comparative microbiome studies in hominids have mainly used a taxonomical approach (*e.g*., only taxa assignment) rather than high-resolution functional metagenomics, ignoring potentials for within bacterial species adaptive evolution.^1,4^

Here, we utilized high-resolution, comprehensive, large-scale metagenomics together with functional and phylogenetic analyses to comprehensively characterize and compare Hominidae microbiomes from two geographically distinct chimpanzee populations (representing different ecology and habitats), the Hadza, and non-indigenous adults and children from rural and urban Tanzania. In doing so, we provide, to our knowledge, the first comprehensive landscapes of the co-evolutionary trajectories of mainly African Hominidae microbiomes and their functional adaptations.

## Results

### Tanzanian hominid microbiomes represent a wide range of primate ecologies and evolutionary landscapes

**Figure 1.**
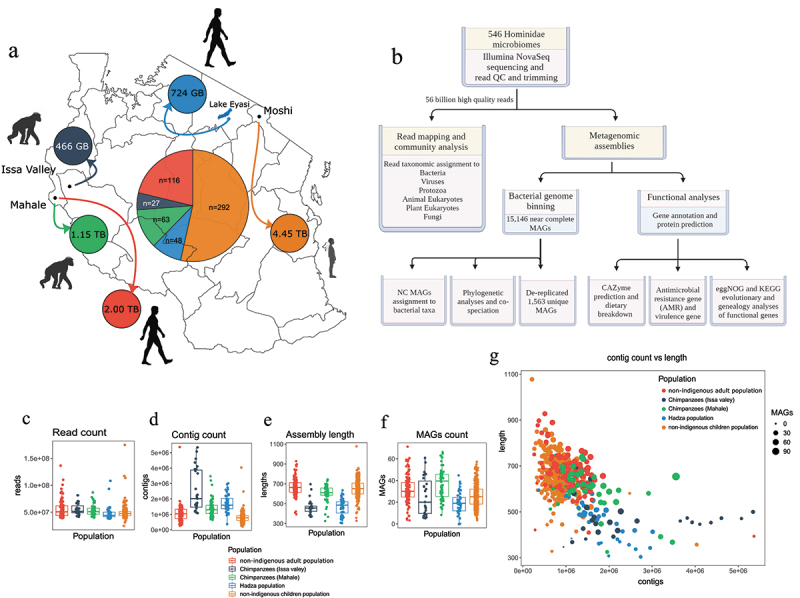
African Hominidae microbiomes from 546 faecal samples from humans and chimpanzees. (a) Summary of the faecal sample collection from the five populations: indigenous Hadza, non-indigenous adult humans, non-indigenous children, Issa Valley chimpanzees, and Mahale chimpanzees, including the sample numbers and locations in Tanzania. Numbers in the pie chart are the sample number within each population, and numbers within each population circle represent the raw output of sequencing data. Arrows indicate the location of the population in Tanzania. (b) Overview of the computational workflow and analyses of all the metagenomes, including the main outputs. (c-f) trimmed and high quality sequencing read pair counts, assembly counts, assembly length, and near-complete (NC) metagenome-assembled genomes (MAGs) outputs of all the Tanzanian faecal microbiomes in this study. A single sample with a very high sequencing output (sample name: DTU2019_MG_976_2015_5E_139–428,976,990 hQ read pairs) was excluded from these plots for visual purposes only, to show the distributions of the remaining samples more clearly. (g) NC MAG and contig outputs from all samples (d-f) compared to each other. Contig counts (x-axis) *vs*. contig lengths (y-axis); size of dots shows the number of MAGs identified in a sample. GB: gigabytes, TB: terabytes, NC: near-complete, MAGs: metagenome-assembled genomes, HQ: high quality.

To investigate the evolutionary signals of Hominidae microbiomes in Tanzania, we studied 546 gut microbiomes from Hominidae (primate) members in Tanzania chosen to represent a wide range of primate hosts reflecting both evolutionary history and different lifestyles (Table S1; Figure 1(a)): (i) 116 and 292 faecal samples from non-indigenous adults from Mahale and school children (P1 to P7, 6–15 years old) from urban Moshi, respectively; (ii) 48 faecal samples from the Hadza, one of the last indigenous hunter-gatherer communities to practise traditional lifestyles despite external pressures, located near Lake Eyasi^1,30^; both sexes were represented in the indigenous populations, where males and females had previously been found to have similar bacterial communities^22^; (iii) 27 and 63 fecal samples from two wild chimpanzee populations (Issa Valley and Mahale, respectively) in the Greater Mahale Ecosystem (GME) area with mosaic landscapes^31,32^; Issa is a mosaic miombo woodland, while Mahale is dominated by Eastern afromontane tropical forest.^31,32^ Both sexes (male and female) and all age groups were represented in the chimpanzee populations.

Microbiomes from feces were DNA-purified, deep-sequenced (average 107 million reads per sample) and analyzed using the same pipeline (Figure 1(b); Table S1; see Methods for details). High-quality trimmed reads were first mapped directly to our custom-made, highly accurate bacterial genome database containing draft bacterial genomes from the NCBI (see Methods for details and similar to,^33^ and 2,836 different bacterial species were identified (Table S2).

To assess higher-resolution (species- and strain-level) microbiome associations with and adaptations to hosts, and subsequently their associated functional adaptation, all reads were assembled into contigs and binned into metagenome-assembled genomes (MAGs). While sequencing outputs were evenly distributed amongst all population samples (Figure 1(c); Table S1), Issa Valley chimpanzees harbored the largest number of microbiome contigs with shorter assembly lengths, followed by the Hadza assemblies (Figure 1(d,e)). The 15,146 near-complete (NC) MAGs were variably distributed between host populations (Figure 1(f); Table S3), with the fewest in Issa Valley chimpanzees, followed by the Hadza, non-indigenous children and adults, and Mahale chimpanzee microbiomes (Figure 1(f)). This graded MAG binning performance within hominid groups was correlated with assembly quality (Figure 1(g)), as seen previously.^34,35^

### Tanzanian hominid microbiomes are richer and more diverse than their western counterparts

**Figure 2.**
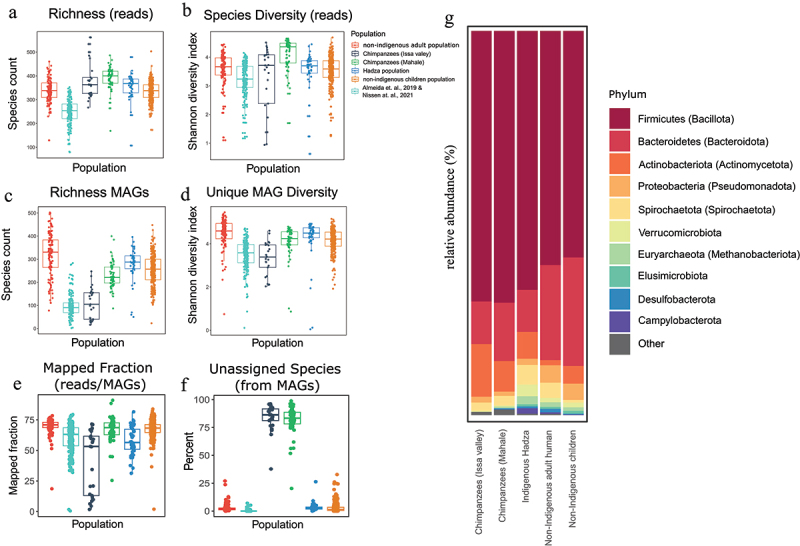
African hominid microbiomes are richer and more diverse than others and separate at the phyla level. (a-b) bacterial richness and diversity of Tanzanian microbiomes compared with previous, mostly western, microbiomes (*n*=72, 92, and 15, from Asia, Europe, and North America respectively)^20^ based on reads mapped directly to our bacterial database. (c-d) bacterial richness and diversity of microbiomes compared with previous, mostly western, microbiomes^20,36^ based on unique MAGs identified in all these microbiomes. (e) The proportions of reads mapped when using our unique MAGs as a database (including the ones from almeida et al., 2019^20^ and Nissen et al., 2021.^36^ (f) the percentage of all identified NC (near complete) MAGs that were taxonomically unassigned to bacterial species. (g) The relative abundance of identified bacterial phyla (MAGs) between populations in all Tanzanian hominid microbiomes. Statistical variations in diversity indices were calculated using a pairwise Wilcoxon test. P-values were corrected for multiple testing, using the BH algorithm (see tables S2 , S3 for full statistical test output).

Chimpanzee microbiomes, especially the Mahale population, harbored significantly richer and more diverse bacterial species than human guts when reads were mapped directly to the bacterial database (Figure 2a,b; Table S2; Wilcoxon test, *p* < 0.05). This was also shown in a recent hominid microbiome study.^10^ When compared to previous human microbiome data,^20,36^ Tanzanian human and Mahale chimpanzee microbiomes had significantly greater richness and diversity (Figure 2(a), B,C,D; Table S2; Wilcoxon test, *p* < 0.05) despite a similar proportion of mapped reads from these samples (Figure 2(e)). A total of 1,563 unique MAGs were identified in human and chimpanzee microbiomes (Tables S3) belonging to 17 phyla (Figure 2(g); Table S3). Of these MAGs, 433 were novel from our taxonomic annotation, with 26 genera that were unassigned. Bacterial MAG species richness and diversity, while varying between populations, were significantly lower in Issa Valley chimpanzees than in the other populations (mean richness: 107 and mean Shannon diversity: 3.30; Figure 2(c,d); Table S3; Wilcoxon test, *p* < 0.05), which were more similar (mean richness: 314, 279, 252, and 230 and mean Shannon diversity: 4.47, 4.24, 4.15, and 4.0 for non-indigenous adults, Hadza, non-indigenous children, and Mahale chimpanzees, respectively, Figure 2(d); Table S3). This was consistent with previously reported similarities between human and wild (not captive) chimpanzees from Congo.^19^ While the lower richness and alpha diversity in Issa Valley chimpanzees may have been due to low MAG binning, richness was high in the Hadza population despite the low number of MAGs (Figure 1(f); Figure 2(c,d)). This is perhaps because Hadza reads could be mapped to MAGs identified in the other human samples (Figure 2(e)). Microbiome richness and alpha diversity in Tanzanian schoolchildren were significantly lower than in non-indigenous adult humans (Figure 2(c,d); Table S3; Wilcoxon test, *p* < 0.05), possibly due to accumulation of more bacterial species over time^37^ and possibly less diverse diet in children.

There were also larger proportions of taxonomically unassigned MAGs in chimpanzees than in other hominid microbiomes (Figure 2(e,f); chimpanzee>Hadza>non-indigenous), suggesting a so far undiscovered number of bacterial taxa in this group, as bacterial databases, including the GTDB database for taxa assignments,^38^ are mainly derived from human guts.

### Core bacterial communities in Tanzanian hominid microbiomes increase with host relatedness

*Homo* and *Pan* hominids are expected to harbor a core microbiome that reflects their common ancestry and physiological demands as well as a unique set of bacteria shared between closely related group members. We determined the core bacterial community in all five populations at the genus level based on reads mapped to the bacterial database as well as our unique MAGs (Tables S8 , S9). Based on mapped reads (before genome assembly) to bacterial databases, the core bacterial community was defined by 91 bacterial genera (Table S8), with *Prevotella*, *Alistipes*, *Bacteroides*, *Escherichia*, and *Ruminococcus* the most abundant, several of which were also identified as core in recent hominid microbiome study.^10^ Based on MAG analyses, only two MAGs were common to all populations (Table S9), but 66 MAGs were common to Issa Valley and Mahale chimpanzees and 50 between all three human populations (Table S9), although there were also distinct differences in higher-resolution microbiomes (based on MAGs) between closely related groups members, e.g., within chimpanzee or human populations. A large proportion of the shared taxa between closely related hosts supports a common ancestry for Hominidae microbiome functional requirements, as the majority of the core bacterial taxa serve common essential functions for metabolism of plant, animal, and microbial substrates (e.g., glycoside hydrolases (GH) such as GH1, GH2, GH3, GH43, GH95 with polysaccharides, celluloses, peptidoglycans, arabinogalactans, and glycoproteins) (Table S7).

### Host relatedness and dietary demands shape microbiome composition, suggesting a mosaic structure mirroring the host population structure

**Figure 3.**
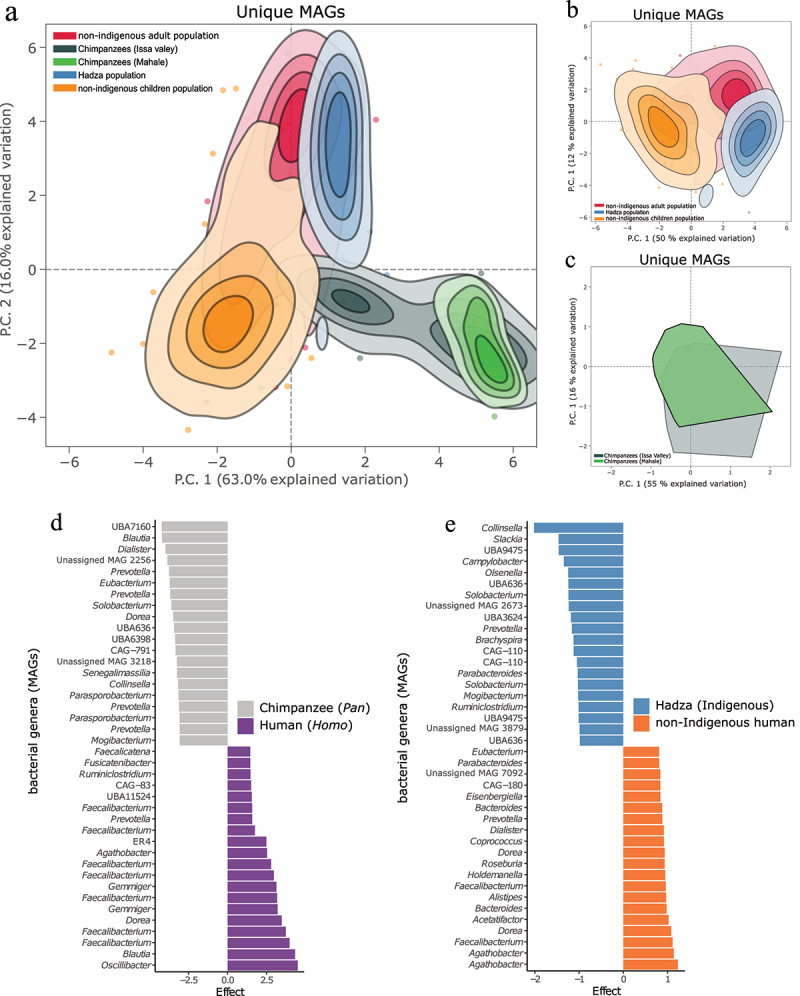
Human and chimpanzee bacterial communities in Tanzania are shaped by host phylogeny and lifestyle. (a) Ordinations of the unique bacterial and archaeal MAGs based on principal component analysis (PCA) visualising that all hominid microbiomes from all five populations are influenced by the hosts. The contours show the microbiome density in each population (each colour) and are truncated at 10% of the peak value. Microbiomes outside this range are represented as individual points with the same colouring scheme. All principal components in the panels are calculated from centred log-ratio (clr)-transformed genome size-adjusted counts, and all contour lines are based on 95% CI (confidence intervals). (b, c) the same bacterial and archaeal MAGs ordination was calculated with only human microbiomes (B) and only chimpanzee microbiomes (c) to show the community similarities within humans and chimpanzees without the effect of larger population size that might mask within-population differences. (d, e) top 20 statistically significant MAGs between human and chimpanzee gut microbiomes (D) and between hadza and non-indigenous human microbiomes (e). Statistically significant genera were adjusted with a benjamini-hochberg false-discovery rate (FDR) correction <0.05 (see figure S3, table S3 for MAG numbers and full list).

**Figure 4.**
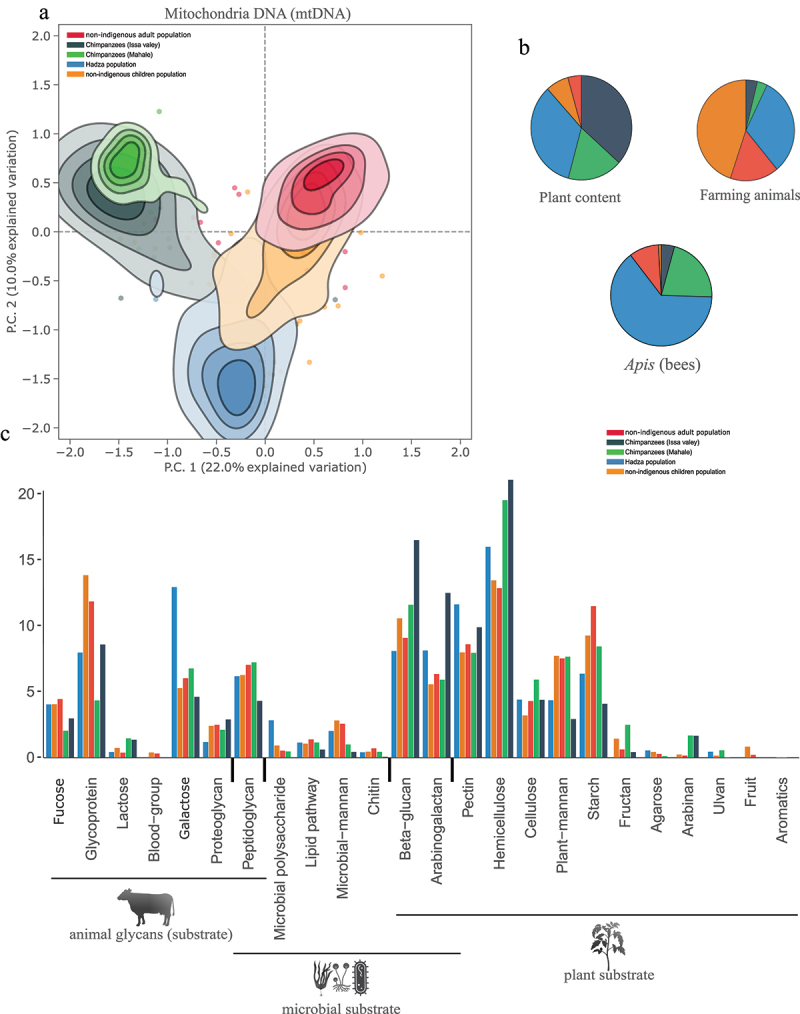
Dietary intake and CAZyme (carbohydrate active enzyme) adaptations mirroring the primate group population and lifestyle. (a) Eukaryotic ordination analysis showing that mitochondrial DNA (mtDNA) from all presumably consumed eukaryotes is shaped by the host primate group and population. The contours show the mtDNA content density in each population microbiome (each colour) and are truncated at 50% of the peak value. Microbiomes outside this range are represented as individual points with the same colouring scheme. All principal components in the panels are calculated from centred log-ratio (CLR) transformed counts, and all contour lines are based on 95% CI (confidence intervals). (b) Distribution of targeted dietary intake (from mtDNA) among the five hominidae populations showing the increased animal diets in humans, plant diets in chimpanzees and hadza, and honey and bees in hadza. All numbers are based on clr-transformed fragment counts and are corrected for the population sample counts. Plant DNA is based on all viridiplantae mtDNA identified in the samples (table S4). Farming animal mtDNA is based on *Bos*, *Gallus* and *sus* species (table S4). *apis* mtDNA is based on all *apis* species mtDNA (table S4). Bar chart of the detailed read counts for each of plant, farming animals and bee mtDNA is presented in figure S1. (c) Abundances of enzymes (CAZymes) putatively targeting all predicted dietary substrates identified in all Tanzanian hominid microbiomes encoded by CAZyme-related genes in all our microbiome MAGs. The relative abundances are calculated from the gene counts encoding a CAZyme function that are then corrected for the total CAZyme gene counts per population, (see methods for details). Each substrate is shown as five bars with different colours representing each of the five populations. The substrates are also grouped according to their potential origin from animal glycans (substrate), microbial and plant substrates.

**Figure 5.**
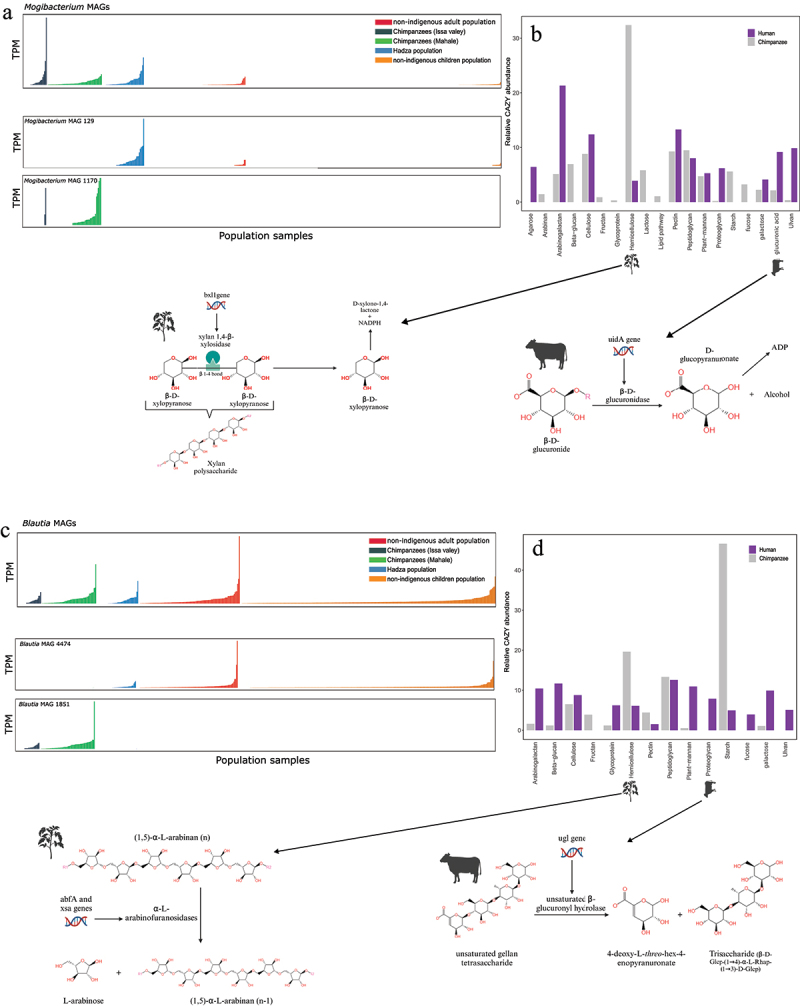
Hominid bacterial and functional adaptations are also observed within one bacterial taxon amongst the populations. (a, c) Bar charts of *Mogibacterium* MAG abundances (a) and *Blautia* MAG abundances (TPM: transcripts per kilobase million) (c). Top plot contains all MAGs assigned to *Mogibacterium* and *Blautia*. The two plots below the total MAGs are representative of the unique MAG abundant in one population or group (*Homo* or *Pan*) over the other. (b, d) Relative abundances of the dietary substrates targeted by CAZyme-encoding genes in *Mogibacterium* MAGs (b) and *Blautia* MAGs (d) showing differences in functional annotations of those CAZymes between *Homo* and *pan*. Relative abundance per sample is calculated as gene counts encoding a CAZyme function and then corrected for the total CAZYme gene counts per population (see material and methods for details). The sample relative abundances are summed in two bars with different colours representing either all human or all chimpanzee microbiomes. Two substrates from each CAZyme substrate collection were selected to show the functional details of the CAZyme-encoding genes and what they target (see material and methods and tables S7 for full statistical test output).

**Figure 6.**
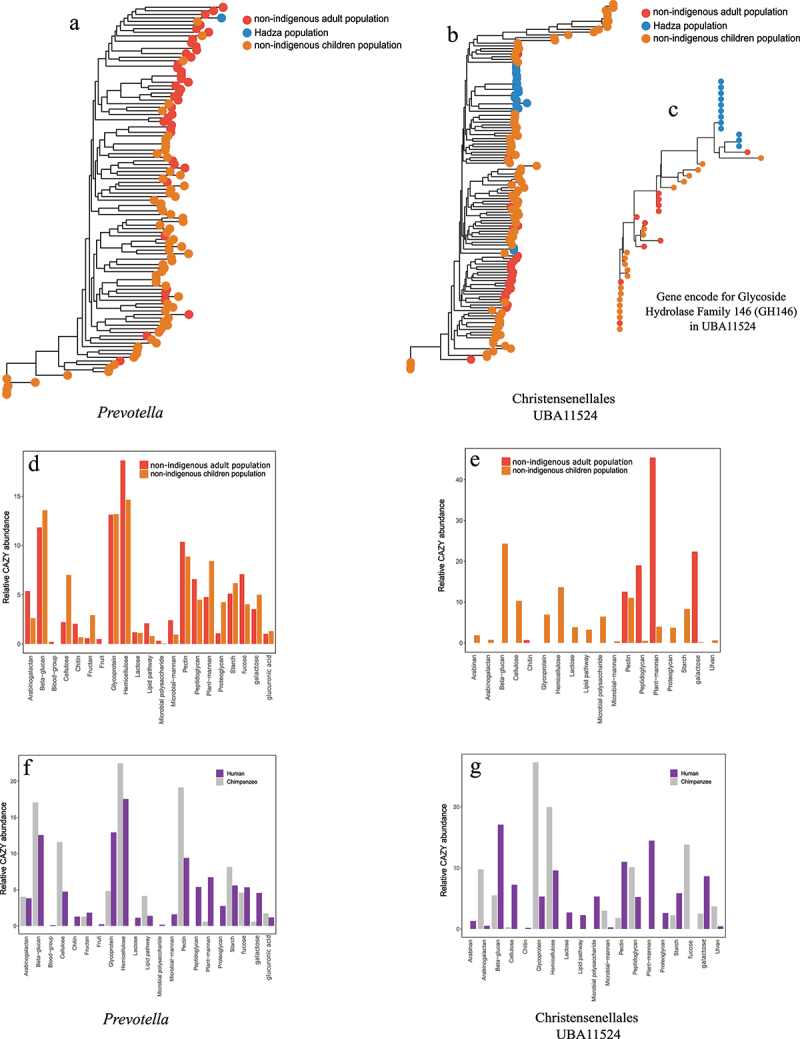
Phylogenetic analyses and co-cladogenesis of selected bacteria and their gene annotations and their functional annotations in dietary degradation within their hominidae group. (a, b) strain-level phylogenetic analysis of *Prevotella* (a) and Christensenellales UBA11524 (B) MAGs visualised by ASTRAL trees. PERMANOVA testing of these trees gave population R^2^ values of 0.055 and 0.093 for *Prevotella* and UBA11524, respectively (*p* < 0.001). The tree tips are coloured according to their hominid host population. (c) SNP tree (maximum likelihood) of a single gene from christensenellales UBA11524 MAGs encoding glycoside hydrolase family 146 (GH146). Gene trees belonging to different gene ontology (GO) functions were tested using PERMANOVA and showed co-speciation with the host population, even at the functional gene level (high population R^2^ values >0.5). Three more genes are also presented in figure S8. (d-g) Relative abundances of the dietary substrates targeted by CAZyme-encoding genes in *prevotella* MAGs (d, f) and christensenellales UBA11524 MAGs (e, g) showing differences in functional annotations of those CAZymes between adult and children microbiomes in the non-indigenous human populations (d, e) and between *Homo* and *pan* microbiomes (f, g). The relative abundance per sample is calculated as gene counts encoding a CAZyme function and then corrected for total CAZyme gene counts per population (see material and methods for details). The sample relative abundance is summed in two bars with different colours representing either non-indigenous adult and children microbiomes (d, e) or all human and all chimpanzee microbiomes (f, g), (see material and methods and tables S7 for full statistical test output).

While all Hominidae members shared several core bacterial genera, there were clear signals from the hominid phylogeny on their gut microbiomes at all taxonomic and genetic levels (Figures 3–6; Figure S1). Human microbiomes were more similar to each other (clustered in ordination analyses and beta diversity) than to their chimpanzee counterparts, harboring similar bacterial taxa (Figure 3). Firmicutes (Bacillota) members were the most dominant bacterial members in all hominid populations. Proteobacteria (Pseudomonadota) and Bacteroidetes (Bacteroidota) were more abundant in non-indigenous populations (adults and children) than in Hadza and chimpanzee guts, while Spirochaetes (Spirochaetota) was most abundant in Hadza microbiomes. Verrucommicrobiota was most abundant in all human microbiomes, and Actinobacteria (Actinomycetota) was more abundant in all chimpanzees and Hadza guts (Figure 2(g)).

At higher resolution, while core microbiome genera (*e.g*., *Prevotella*) were common in all gut microbiomes, differential abundance analyses of unique MAGs showed significant variations between primate guts (Figure 3). Certain taxa were significantly more abundant in human or chimpanzee guts (Figure 3(d), Figure S3), e.g., members of *Faecalibacterium* and *Gemmiger* in humans and *Blautia* and *Mogibacterium* in chimpanzees. Similarly, distinct subsets of bacterial taxa also significantly characterized the microbiomes of Hadza and non-indigenous human populations (Figure 3(e) Figure S3), e.g., members of *Faecalibacterium* and *Alistipes* in non-indigenous humans and *Brachyspira* in Hadza. A full list of all differentially abundant taxa and their MAGs is provided in Figure 3, Figure S3 and the Supplementary Information. The bacterial communities were more similar between non-indigenous adults and children and between Issa Valley and Mahale chimpanzee populations (Figures 3(b,c); Figure S1). This mosaic structure of bacterial communities in hominid guts in Tanzania suggests a large shared community of gut bacteria but also enrichment of a subset of bacteria that might have co-speciated with the host group for a specific function. Thus, while the same taxon might exist in both human and chimpanzee populations (*e.g*., *Blautia*), it belongs to different genomes (MAGs) in the two populations.

### Host relatedness, ecology, and lifestyle also structure non-bacterial communities

We also characterized the non-bacterial components in the primate microbiomes by mapping all high-quality trimmed reads to several DNA virus, bacteriophage, fungus, parasite and protozoa databases (see Supplementary Information, Table S4). Hominid microbiomes also harbored very rich microbial and eukaryotic communities: 264 DNA viral species, 102 large bacteriophages, 876 fungal species (coverage > 50) and 312 protozoa and parasitic species (Figure S1 ; Table S4).

The viral communities showed clear ecological patterns, with Hadza microbiomes enriched in mammalian Mastadenovirus (Adenoviridae),^39^ while chimpanzee microbiomes harbored aquatic-associated Prasinovirus and Alphapapillomavirus, suggesting environmental acquisition (Figure S1). Similarly, the jumbo phage community (jumbo phage database adapted from^40^ with similar jumbo phages in chimpanzee populations (e.g., vB_ShiP-A7 and vB_Eco4-M7 phages) compared to Hadza and urban children and adults (Figure S1), underscoring shared ecological niches.

Non-bacterial community differences also reflected dietary and lifestyle influences, with edible fungi like *Tricholoma* and *Morchella* more prominent in chimpanzee and Hadza microbiomes, while other taxa such as *Termitomyces* and *Cladosporium* were more abundant in non-indigenous human populations (Figure S1, Table S4). Parasites and protozoa further emphasized the role of host lifestyle and environment, with *Schistosoma mansoni* prevalent in children likely due to waterborne transmission,^41,42^ and *Enterobius vermicularis* likely reflecting direct human-to-human contact in communal living settings (Table S4). These findings demonstrate that non-bacterial communities are shaped by host-related factors and ecological context, contributing to the functional and evolutionary diversity of the microbiome.

### Dietary predictions differ between hominid populations

The genetic reservoir of the Tanzanian Hominidae microbiome was also largely mirrored by host phylogeny and lifestyle. Using microbiome DNA reads, we examined dietary intake differences by mapping Tanzanian microbiome DNA to a mitochondrial DNA (mtDNA) database (including Viridiplantae mtDNA), and 1,959 plant and animal taxa were identified. Plant and non-host animal mitochondrial DNA clustered according to the host population in ordination analyses (Figure 4(a)). Plant mtDNA fragments were more abundant in chimpanzee and Hadza gut microbiomes (Figure 4(b); Figure S1), while mtDNA from livestock (*Bos*: cow, *Sus*: pigs, and *Gallus*: chicken) were more abundant in all human microbiomes (Figure 4(b); Figure S1), consistent with a plant-dominant diet for chimpanzees and Hadza and meat consumption by both indigenous and non-indigenous humans. Bee (*Apis*) and bee-derived mtDNA was also markedly more abundant in Hadza gut microbiomes (Figure 4(b); Table S4), possibly reflecting the high honey intake by the Hadza.^43^ Bee DNA was also abundant in Mahale chimpanzee guts (Figure 4(b)), where honey and bees were described to be part of their dietary intake.^44^

### Hominidae domesticated their gut microbiomes for increased fitness and lifestyle adaptation, displacing or functionally adapting bacterial taxa

As Tanzanian hominid microbiomes showed strong signals of host phylogeny, population and dietary intake, we investigated whether and how those signals were also related to function by examining the functions of the identified microbiomes. Hominid microbiomes harbored 7,943,477 CAZyme-encoding genes^45^ (in four categories: AA, auxiliary activities; GH, glycoside hydrolases; PL, polysaccharide lyases; CE: carbohydrate esterases), 44% of which could be assigned at least one putative function (Table S6; Table S7). A total of 4,295 contigs (with 10,411 genes) had both bacterial taxonomic assignment and CAZyme functional hits. KEGG (Kyoto Encyclopedia of Genes and Genomes) functional analyses showed enrichment for functional cellular processes, environmental information processing, genetic information processing, human diseases, metabolism, and organismal system pathways in our African hominid microbiomes compared to other KEGG pathways (Figure S2).

We first examined dietary metabolism (CAZymes) associated with individual microbiomes and identified differences in functional capacity and pathways (similar to^46^ between and within the primate groups (Table S7). All hominid microbiomes harbored enzymes predictive of omnivorous food degradation (*e.g*., fruit, lipids, chitin, and arabinogalactan) (Figure 4(c)), consistent with their omnivorous diets.

However, each individual primate group was also enriched for enzymes targeting either animal-derived or plant-derived diets (Figure 4(c); Figures 5(b,d); consistent with mtDNA, Figure 4(b)). Chimpanzees harbored microbiomes with increased capacity for digesting plant-derived substrates such as complex polysaccharides (Figure 4(c), Table S7). Human microbiomes harbored bacteria enriched for breakdown of animal-based complex polysaccharides such as glycoproteins and proteoglycans (Figure 4(c), Table S7). These functional adaptations were attributable to host-specific bacteria (Figures 3–6). However, this adaptation was also evident within bacterial taxa. Thus, *Mogibacterium*, specifically its chimpanzee-abundant bacterial MAGs (Figures 3(d), 5(a), Table S7), had different predicted functions in humans and chimpanzees. CAZyme-related genes in chimpanzee-associated *Mogibacterium* MAGs encoded the enzyme xylan-1,4-β-xylosidase, which targets the β-1–4 bonds of β-D-xylopyranose molecules forming xylan polysaccharides, a primary constituent of hemicellulose from plants^47^ (Figure 5(b); Table S7; significance is based on bootstrap resampling approach (10,000 replicates) with 95% CI excludes zero). While *Mogibacterium* was less abundant in humans (Figure 5(a)), it also demonstrated a functional shift toward animal-derived substrate degradation (e.g., proteoglycan and glucuronic acid) (Figure 5(b); Table S7); particularly in Hadza guts (Figures 3(d), 5(a)), it encoded β-glucuronidase, enzymes pivotal for targeting animal-based glycans such as glucuronides (Figure 5(b); Table S7; bootstrap 10,000 replicates with 95% CI excludes zero).

Similarly, while *Blautia* was present in all hominid microbiomes, unique *Blautia* MAGs were differentially abundant in humans and chimpanzees (Figures 3(d), 5(c); Figure S3). In chimpanzee guts, *Blautia* MAGs degraded large polysaccharides from plants, particularly starch, cellulose, and hemicellulose (Figure 5(d)), such as through abfA and xsa in chimpanzee *Blautia* MAG 1851, which encode α-L-arabinofuranosidases that produce L-arabinose from plant cell walls (arabinans in hemicellulose) (Figure 5(d); Table S7; bootstrap 10,000 replicates with 95% CI excludes zero).^48^ Human-specific *Blautia* MAGs (Figure 5(c,d)) were also involved in plant degradation (arabinogalactan and β-glucan) but also harbored more genes involved in animal tissue degradation (proteoglycans) (Figure 5d). For example, genes in human *Blautia* (MAG 4474) encode lysozymes and unsaturated β-glucuronyl hydrolases, which break down large animal peptidoglycans and unsaturated poly-saccharides with a terminal-linked unsaturated uronic acid and other proteoglycans into monosaccharides (Figure 5(d); Table S7; bootstrap 10,000 replicates with 95% CI excludes zero).^49,50^

Other examples of functional specialization in different hosts were observed, for instance *Dorea* MAGs (NODE2822 and NODE 2784) and *Faecalibacterium* had separate functions in human and chimpanzee gut microbiomes (Figure S4, Figure S5, Table S7). *Alistipes* had separate functions in Hadza and non-indigenous microbiomes (Figure S4, Figure S5, Table S7). See Supplementary Results for details.

However, *Brachyspira*, an abundant bacterium in Hadza microbiomes (Figure 3(e)), belonged to MAG 619 (Figure 3(e); Figure S3) but harbored separate gene functional profiles between non-indigenous and indigenous guts (Table S7). Specifically in Hadza microbiomes, *Brachyspira* harbored genes involved in β-1,3–1,6-galactose and α-N-arabinofuranosidase production (GH35, GH43) (Table S7), which cleave or hydrolase plant-derived pectins and arabinogalactans, and could also be found as plant residues in honey in smaller content.^51,52^ These glycoside hydrolases are also linked to honey polysaccharide degradation in honeybees.^51,52^ The increased abundance of those glycoside hydrolases in Hadza guts is consistent with their high dietary intake of plant and honey. A full list of all predicted CAZymes and their substrates is provided in Table S7, with significance based on bootstrap 10,000 replicates with 95% CI excludes zero.

Our compositional and functional analyses of the Tanzanian hominid microbiomes revealed not only bacterial taxa displacement of unique MAGs with specialized enzymatic capabilities between the different host populations but also that shared bacterial taxa perform different functions to accommodate host dietary needs. This suggests that gut microbes adapt to diet at a very high resolution, *i.e.*, different degradative capabilities for different genes within the same genus.

### Evolutionary adaptations in hominid microbiomes highlight deep co-speciation and functional differences beyond taxa displacement

We next investigated the depth of the host signal effect on bacterial communities at the MAG strain level to evaluate the degree of co-cladogenesis (in this context, parallel evolutionary divergence of hosts and their associated microbial taxa) and phylosymbiosis in Tanzanian Hominidae members. Unique MAGs with the highest density of MAG counts across samples (>100 MAGs within the species) were identified. Due to the strong host signature on gut microbiomes, most of the unique MAG were primarily found in either human or chimpanzee microbiomes (Figures 3 and 6, Figure S5), permitting strain-level comparisons only between bacterial MAGs found primarily in humans or chimpanzees, even when belonging to core bacterial taxa.

We identified 14 MAG species present in >100 samples (Table S10; C1-C14) and included one additional MAG species of interest (Table S10; C15). To investigate how the host population affected strain separation within these species, we created a phylogenetic tree of each MAG species (two in Figure 6(a,b); all in Figure S6). All trees were subjected to PERMANOVA analyses, where the resulting R^2^ values describe how well host populations explain the variation found in the trees. On average, 5% of variation in the trees could be explained by the host populations. *Prevotella* and Christensenellales UBA11524 phylogenetic trees and analyses showed a higher level of co-cladogenesis with the host population (R^2^ 0.055 and 0.093, respectively), where the bacterial strains from non-indigenous humans (adults and children) were more similar to each other than to the Hadza strains (Figure 6(b)). This was also true for several other MAG species where we tested community co-speciation with the host phylogeny (Figure S6, Table S10).

To confirm that several bacterial strains were unique to the Tanzanian hominid microbiomes, co-speciated bacterial strains in the microbiomes were compared to strains from independent microbiome datasets (from.^14,20,36^ The comparative analyses showed that our Tanzanian homonid strains were phylogenetically distant from bacterial taxa acquired from those independent human microbiome datasets (Figure S7).

We next examined if any genes or groups of genes were also shaped by the host population and exhibited codivergence (or co-evolutionary congruence) and how those genes contributed to the phylo-symbiotic relationships between host and strain. From the Christensenellales UBA11524 MAG species, we identified 4,436 orthologous genes, of which 3,398 genes were amenable to PERMANOVA and 1,308 of which could be functionally annotated using biological process gene ontology (GO) terms (Figure S8). Four genes involved in pectin breakdown (GH146) and stress (redox) showed high levels of host-gene specificity (R^2^ values > 0.5) (Figure 6(c); Table S7). The phylogenetic differences correlating with host population and lifestyle suggest that in addition to adaptation of enzyme abundances, adaptation can also occur through mutations in genes abundant across different host populations. To finalize the evolutionary adaptation analyses of the co-speciated taxa, we next performed functional analyses on those selected taxa and genes. The core microbiome member *Prevotella* showed distinct functional profiles in humans and chimpanzees. In humans, *Prevotella* was involved in animal proteoglycan and glycoprotein break down, encoding β-N-acetylhexosaminidase targeting galactosamine (Figure 6(f); Figure S4; Table S7; bootstrap 10,000 replicates with 95% CI excludes zero), and α-galactosidases that cleave plant mannan (Figure 6(f); Figure S4; Table S7). Conversely, *Prevotella* encoded enzymes that degrade cellulose, hemicellulose, and pectin in chimpanzee guts (Figure 6(f), Figure S4, Table S7; bootstrap 10,000 replicates with 95% CI excludes zero). However, this functional variability was only present between *Homo* and *Pan* hosts and not between more closely related hosts with similar lifestyles such as non-indigenous adults and children (Figure 6(d)), as the functional profiles became more similar between these two groups. Christensenellales UBA11524, while common to all Hominidae microbiomes, showed host-microbiome co-specificity, not only between human (plant-degrading, e.g., pectin and plant mannan) and chimpanzee (animal protein-degrading, *e.g.*, glycoprotein) microbiomes (Figure 6(g)), but also within human microbiomes. Christensenellales UBA11524 was more involved in degrading plant mannan (complex plant polysaccharides) in non-indigenous adults, and beta-glucans and hemicellulose as plant substrates, as well as lactose and glycoproteins (animal glycans) in non-indigenous children (Figure 6(e); Table S7; bootstrap 10,000 replicates with 95% CI excludes zero). Such variation within and between closely related hosts suggests a high level of adaptation covering various layers of host relatedness (between humans and chimpanzees, as well as within non-indigenous humans).

**Figure 7.**
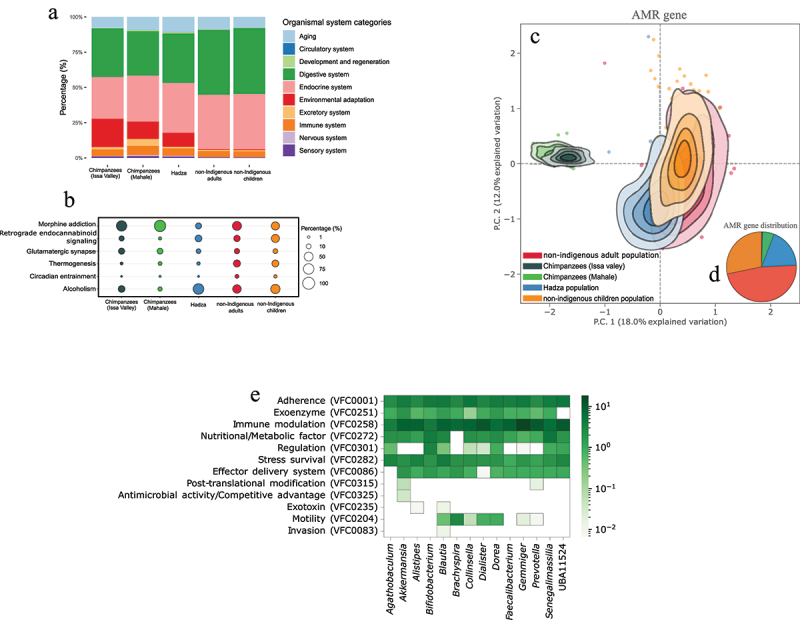
Non-dietary functional annotations (KEGG, antimicrobial resistance, and virulence genes) in Tanzanian hominid microbiomes show adaptive responses to host phylogeny and population. (a, b) pathway annotation based on KEGG orthology analysis within the organismal systems category between the five hominid populations (a). The fractions of each KEGG pathway represent the relative abundance of the genes involved in the assigned function. More detailed KEGG pathway abundances were also investigated that were involved in the nervous system, environmental adaptation, and substance dependence pathways, which showed variations between the five Tanzanian hominid populations (b). Circle sizes in (b) indicate the relative abundance of a KEGG function in the corresponding population, and colours represent different populations. (c, d) resistome ordination analysis showing that antimicrobial resistance genes (ARGs) are primate host influenced (c), with non-indigenous humans harbouring the majority of the microbiome ARGs (d). The contours in (d) show the resistome density in each population (each colour) based on the 100 AMR genes with highest relative abundance and highest variance across the population. Resistomes outside this range are represented as individual points with the same colouring scheme. All principal components in the panels are calculated from centred log-ratio (clr)-transformed genome size-adjusted counts, and all contour lines are based on 95% CI (confidence intervals). (e) Presence of virulence genes in 14 differentially abundant bacterial MAGs across all our Tanzanian microbiomes. All virulence occurrences from all MAGs are included in the supplementary information, focusing here on our differentially abundant bacteria. All virulence genes are represented by their virulence factor category (left side), and the relative abundance of those genes in each MAG are represented in log-transformed scale within the heatmap and based on our tailored zoonotic VFdb; refer to material and methods.

We extended our functional predictions with KEGG orthology analysis,^53^ which revealed a pronounced increase in certain physiological categories over others in different hominid groups (Figure 7(a,b)). Proteins predicted to be involved in cellular processes, environmental information, human diseases, and organismal systems were differentially abundant between the hominid group microbiomes (Table S11). Genes involved in environmental adaptations significantly varied between human and chimpanzee KEGG functional profiles in microbiomes (Figure 7(a,b)), mainly because of pathogen interaction genes (KEGG pathway ko04626, 86 genes), circadian rhythm (KO 04710, 28 genes), and thermogenesis genes (ko04714, 188 genes).^53^ Non-indigenous adult and child microbiomes contained more bacteria involved in host circadian rhythm, regulating host thermogenesis, or producing more heat during dietary breakdown compared with Hadza and chimpanzee guts (Figure 7(a,b)), perhaps due to a non-indigenous lifestyle characterized by a more climate-controlled environment and carbohydrate-rich diet.

These results suggest that these microbes can adjust to changes in their environment, especially varied and complex diets. Such flexibility is integral to maintaining gut homeostasis, as these microbes must rapidly adapt to dietary fluctuations.

### Non-indigenous urban Tanzanians harbour more antibiotic resistant bacteria and distinct virulence genes

We also characterized hominid resistomes by mapping microbiome reads to the ResFinder database,^54^ and 21 and 1,003 AMR (antimicrobial resistance) classes and genes were identified, respectively (Table S5). Few antimicrobial resistance genes (ARGs) were present in chimpanzee gut microbiomes, but they were abundant in non-indigenous human guts with distinct gene pools in all human microbiomes compared with their non-human hominid relatives (Figure 7(d)). Within hominid AMR classes, ARGs encoding resistance to tetracycline were the most abundant and ARGs to colistin were the least abundant (Figure 7(c) ; Table S5). Human guts contained a higher abundance of genes encoding resistance to beta-lactams, macrolides, and lincosamides, while chimpanzee guts were enriched for aminoglycosides, macrolides, and beta-lactams (Table S5). Beta-lactam ARGs were more abundant in children and macrolide ARGs were more abundant in adults, while aminoglycoside ARGs were the most abundant in Hadza microbiomes (all after tetracycline ARGs). At the ARG level, there was clear separation in resistome composition and structure between humans and chimpanzees (Figure 7(c)), mainly due to higher abundance of different VanC3XYgenes in chimpanzee microbiomes and tet (Q), tet(W) and bla genes in human guts. In humans, rmtG, rmtD, rmtG, rmtF, and tet(44) were overrepresented in Hadza microbiomes and sul2, tet(B), and blaOXA in non-indigenous human guts (Figure 7(c); Table S5). There was no difference in specific ARG load between adults and children.

tet(Q) and tet(W) have been identified as conjugative elements in several different bacterial species, but mainly in *Bacterioides* spp., *Prevotella* spp., and some other related anaerobic bacteria.^55^ Sul2 and tet(B) are associated with plasmids transmitting between Gram-negative bacteria.^55,56^ *bla*OXA is a very large group of beta-lactamases often found in plasmids but also intrinsic to some species.^57^ Thus, the increased abundance of these ARGs in urban and rural human microbiomes suggests exposure to and selection by antimicrobial agents.

vanC3 is intrinsic to *Enterotoccus flavescens*,^58^ and its higher abundance in chimpanzees is most likely associated with a higher abundance of this species. The high abundances of rmtG, rmtD, and tet(44) in Hadza is more difficult to explain. The rmt enzymes encode 16S methyltransferases that cause resistance to all aminoglycosides, and they are widely distributed among clinical Enterobacteriaceae^59^ and were first identified on a transmissible genomic island in *Campylobacter fetus*.^60^ There are no data on antimicrobial usage in the Hadza, but they are not entirely isolated, and it cannot be excluded that the existence of these ARGs is due to contamination from tourists or other visitors or through the use of antibiotics. Another possibility is that the Hadza are naturally exposed to environmental aminoglycosides. Of note, the Hadza had a very high abundance of reads mapping to *Streptomyces*, the genera containing aminoglycoside-producing organisms.

The presence of ARGs in the Hadza and wild chimpanzees may suggest that resistance mechanisms have diversified differently from non-indigenous human microbiomes. Over time, horizontal gene transfer – the ability of bacteria to share genetic material – could have spread these resistance genes throughout various bacterial populations, even those residing in human and animal guts.

Finally, we detected 365,553 virulence factor genes in 14 virulence factor classes (Table S12). Sixteen bacterial MAGs contained 32.4% of all identified virulence genes (Table S12), largely belonging to common pathogenic bacteria members in *Escherichia*, *Klebsiella*, *Enterobacter*, *Pseudomonas*, *Haemophilus*, and *Citrobacter* (Table S12; Figure S9). We focused our virulence gene detection to only differentially abundant bacteria between humans and chimpanzees (Figure 3(d)), and the majority of these genes were involved in adherence, immune modulation, and stress survival (Figure 7(e)). While the genes involved in those virulence classes were distributed amongst those genera (Figure 7(e)), *Gemmiger* MAGs, a human bacterium, harbored the majority of immune modulation virulence genes (Figure 7(e)), particularly capsular polysaccharide (CPS) genes involved in antimicrobial activities.^61^ *Blatuia* (specifically MAG 1851 in chimpanzees, Figure 5(c)) contained more genes involved in stress survival, particularly caseinolytic protease genes, such as ClpC and ClpP (Table S12). Clp proteases and chaperones are involved in stress responses, particularly heat regulation and virulence.^62^ This *Blautia* MAG was significantly more abundant in chimpanzee guts (Figure 5(c)), who are subjected to a less heat-regulated environment than humans. Motility-related genes were only abundant in *Blautia*, *Brachyspira*, *Collinsella*, *Dialister*, and *Dorea* (Figure 7(e)), all of which were differentially abundant between hominid microbiomes (Figure 3). Motility-related genes are often linked to bacterial virulence,^63,64^ indicating that motility-related virulence is also influenced by the hominid host.

## Discussion

Here we show that, while all hominid gut microbiomes in Tanzania, including indigenous Hadza, have similarly high microbial diversity compared with their westernized counterparts, there were distinct gut microbiome differences according to cultural and geographical context. Our exhaustive sequencing effort provides pivotal data on gut microbiomes from historically underrepresented regions, refining our understanding of the gut microbiome landscape in Tanzanian hominids and the indigenous Hadza population.

A set of bacterial taxa were largely adapted to specific primate population requirements in Tanzanian Hominidae gut microbiomes. They shared common bacterial ancestry, as they all originated from at least the same genus in MAGs. However, bacterial taxa had also diversified to serve specific functions in specific guts, with a number showing co-cladogenesis with the host populations. This observation suggests that these patterns did not arise from recent microbial evolutionary processes, as would have been the case if the patterns arose via microbial lineage splitting concurrent with host lineage splitting.^65,66^

Two recent studies^23,24^ and others before (e.g.,^15^ reported an increase in gut microorganism diversity in the Hadza compared with other microbiomes, mainly non-African, and a Western lifestyle seems to diminish gut microbiome diversity. These reports concluded that these microbes in Hadza can better adjust to environmental changes, especially in response to varied, complex diets. Such flexibility is integral to maintaining gut homeostasis, as these microbes need to rapidly adapt to fluctuations in dietary nutrients. However, none of those reports compared Hadza microbiomes to their homogenous nearby Tanzanian microbiomes, for example to non-indigenous Tanzanian microbiomes, which were similarly richer and more diverse than their United States or European counterparts.

Similar to previous studies^5,12,13^ we found that the composition of the gut microbiome to a very large extent is driven by the capabilities of these bacterial taxa to degrade the host diet. However, importantly, we also found that within certain taxa the bacteria have adapted to be capable to perform niche-specific functions. This shows the evolutionary adaptability of certain bacterial taxa, performing variable functions across different host microbiomes. It also shows that investigating bacterial taxa diversity, for example using 16S profiling, is not sufficient to understand the complex functionalities of the gut microbiome, and that extending sequencing to the entire bacterial genomes is necessary.

The presence of members of Christensenellales in our Tanzanian microbiomes highlights their heritability, as previously observed in human twin studies.^2,67,68^ Our findings provide further evidence for this strong genetic influence, as the population-specific clustering and functional divergence of Christensenellales reflect the interplay between host genetics and environmental factors. While previous reports showed that Christensenellales showed increased microbial biomass and alteration in physical activity in mice,^69^ the detailed contributions of Christensenellales members to host physiology remains largely unknown.^69^ Our study however showed that members of one genus in Christensenellales, UBA11524, were more adapted to degrade plant-based substrate in human guts compared to chimpanzee guts. Even within human guts, functional variations were observed between adults and children. This functional variation and heritability in our Tanzanian microbiomes explains the evolutionary importance of Christensenellales in shaping host-microbiome co-adaptations and functional specialization across different host populations. These findings contribute to the growing understanding of how heritable microbiome members adapt to meet host-specific physiological and dietary demands, further strengthening the hypothesis of microbiome-host co-evolution.

The Tanzanian gut fungal communities were dominated by mainly 30 species, accounting for 90% of the total fungal abundance. Of which, edible fungi such as *Tricholoma* (yellow knight) and *Morchella* (morel mushroom)^70^ reflect wild foraging practices, with the latter being known for its culinary value. Additionally, plant pathogens like *Alternaria alternata* and *Puccinia arachidis* ^71,72^ highlight environmental and agricultural influences, potentially linked to contact with crops.

The detection of *Termitomyces* fungi in the non-bacterial communities of the Hadza and chimpanzee microbiomes underscores the ecological and dietary significance of this genus in African environments. *Termitomyces* species, the fungal symbionts of the African fungus-growing termites,^73^ are widely consumed across the continent due to their environmental abundance,^74^ with average annual household consumption reaching 30–35 kg.^70^ In Tanzania, *Termitomyces* species such as *T. titanicus*, *T. letestui*, and *T. eurhizus* are not only dietary staples but are also utilized in traditional medicine for treating intestinal ailments, including ulcers and constipation.^75^ The presence of *Termitomyces* and other fungal species in the gut microbiomes of Tanzania may reflect both dietary intake and potential functional contributions to gut health, suggesting an ecological and evolutionary role for these fungi in shaping host–microbe interactions.

The high abundance of *Schistosoma mansoni* in children aligns with previous reports that this parasite is particularly prevalent among children in Africa, with reported high infection rates due to frequent contact with contaminated water sources and prevalence rates among preschool-aged children in some regions reaching up to 90%.^76^ The presence of *Giardia intestinalis* and *Trichuris trichiura* in non-indigenous adults likely reflects exposure to contaminated food and water, potentially exacerbated by urbanization-linked sanitation issues. The predominance of *Enterobius vermicularis* in the Hadza, a parasite transmitted directly between humans, highlights how communal living and frequent interpersonal contact can facilitate parasite spread. This finding may project an evolutionary trade-off in hunter-gatherer populations, where the health impacts of parasitism are potentially mitigated by a more diverse and robust microbiome.

It was interesting to note the high abundance of *Apis* mitochondrial DNA in the Hadza population and related bacterial adaptation at the single gene level to honey and plant degradation. Honey is especially valuable to the Hadza, who have established a mutualistic relationship with Greater Honeyguide (*Indicator indicator*) birds to guide them to honey nests.^43^ Honey also forms a significant part of the Hadza diet, constituting as much as 15% of the calorie intake^77^ compared, for example, to 0.12% in Danes (FAO-STAT - https://www.fao.org/faostat/en/#data/FBS.). Honey appears to have a very significant impact on the ecology of the Hadza gut microbiome, which would be interesting to examine in other societies with high honey consumption.^77^ We also observed an above average abundance of reads mapping to bee DNA in the chimpanzee from Mahale. A number of chimpanzee communities have been described to consume honey (e.g.,^78^ and communication with the Mahale community, however, further studies are needed to establish chimpanzee honey intakes.

On resistome, the higher abundance of ARGs in non-indigenous human populations compared to wild chimpanzees is consistent with studies highlighting the impact of human activities on resistome profiles.^19^ Apes in captivity exhibit higher ARG abundance and diversity than their wild counterparts, likely due to increased exposure to human-associated bacteria and antibiotics in managed care settings.^19^ The detection of ARGs in the Hadza microbiome, despite limited direct exposure to antibiotics, suggests that natural environmental reservoirs and traditional lifestyles contribute to resistome composition. Studies have identified ARGs in environments without anthropogenic antibiotic use, indicating that resistance genes are ancient and widespread, maintained through natural microbial interactions.^79^ The presence of ARGs in wild chimpanzees and the Hadza highlights the potential for cross-species spillover, where resistant bacteria could transfer between humans and wildlife, posing risks to conservation and public health.

The presence of a distinct subset of bacterial taxa in Hadza gut microbiomes related to their specific lifestyle raises the prospect of ‘microbiome fingerprinting’ in guts, where an individual’s or population’s lifestyle, and perhaps even dietary history, could potentially be determined through comprehensive gut microbiome analysis. This study provides some of the first empirical evidence of such host domestication of the gut microbiome (similar to,^80,81^) which could be of importance to diverse fields like anthropology, forensics, and personalized medicine.

Microbiome adaptation is not a uniform process but one that involves dynamic displacement or functional adaptation between and within bacterial taxa. Here, we showed microbiome–host interactions at strain level to demonstrate host-related adaptive mechanisms. These mechanisms were not only responsible for selecting particular bacterial strains but also appeared to shape bacterial functions toward host-specific needs, leading to functional divergence between human and chimpanzee gut microbiomes.

Our discovery of resistomes across hominid species offers a striking perspective on the complex evolutionary strategies employed by microorganisms in the face of antibiotic use. The surprising prevalence of antimicrobial resistance in seemingly isolated communities, such as wild chimpanzees and the Hadza, prompts reevaluation of our assumptions about antibiotic resistance origins, dissemination, and its potential implications across different ecosystems.

A recent study^10^ showed similar output into functional host-specific adaptations in hominid gut microbiomes, like reduced microbial diversity in humans compared to apes. They linked this loss also to lifestyle changes, including higher antibiotic use and dietary shifts in high-Human Development Index (HDI) regions, as we did. While their study focused on non-human apes, African rural villagers, and westernized humans, our inclusion of Hadza populations showed a unique perspective on microbiomes shaped by traditional hunter-gatherer lifestyles. For example, we observed that *Prevotella* species, which Rühlemann et al.^10^ identified as dominant in non-westernized human populations, were enriched in Hadza microbiomes, further supporting their role in plant-rich diets and traditional lifestyles. Our study expanded on their findings by characterizing functional adaptations of *Prevotella* through carbohydrate-active enzyme (CAZyme) analysis, revealing key enzymatic pathways associated with plant polysaccharide metabolism in Hadza microbiomes.

Rühlemann et al.^10^ reported disrupted co-phylogeny patterns in human microbiomes, reflecting a deviation from evolutionary host-microbe relationships, which we corroborated by identifying distinct not only taxonomic but functional displacement driven by host-related factors such as dietary intake, lifestyle, and ecology. All of which confirm the coevolutionary adaptations of the hominid gut microbiome, shaped by host genetics, ecology, and lifestyle, while our study adds depth by including Hadza microbiomes, CAZyme analysis, pathogenicity and non-bacterial taxa, and allows to study the effect of lifestyle and evolution on the microbiome without the geographical effect as all samples were from Tanzania.

The observed co-speciation and intricate co-evolution between hosts and their gut microbiomes suggest a two-way ‘adaptive feedback loop’.^82^ While host factors shape the gut microbiome, the microbiome in return could influence the host’s evolutionary trajectory. It may be that some human or primate traits were developed not solely by traditional genetic evolution but through a more conservative mutualistic process involving their microorganism partners.

Microbiome-driven evolution could suggest that the gut microbiome, with its extensive genetic material and rapid rate of evolution, could potentially play a much larger role in host adaptation and survival than previously believed. The gut microbiome acts as an ‘auxiliary genome’, providing the host with an additional layer of genetic diversity and adaptability, responding to environmental changes faster than the host genome and thus offering a survival advantage.

### Limitation of the study

This study was designed to investigate the microbiome adaptations to various primate lifestyles in an understudied population in Africa (Tanzania) using metagenomics sequencing. Although we cover a large population of Hominidae in Africa and used standardized methods to compare all microbiomes, there are limitations to this study: populations have imbalanced sample sizes, particularly modest in the Issa Valley chimpanzee and Hadza groups, which arise from challenges inherent in collecting uncontaminated wild chimpanzee feces and numerous Hadza samples without major disturbance. This also meant a lack of major comparisons of equally comparable metagenomes (high quality) from wild chimpanzees and hunter-gatherers. Another major limitation is the lack of functional assays to complement our sequencing output, as this study relies heavily on *in silico* data, similar to most microbiome studies. The difficulties of working with culturable microbiome bacteria, and subsequently functional assays, is a well-known challenge, however, we hope in our follow-up studies we will be able to add several functional assays, particularly linked to diet degradation and CAZyme function on a selected bacterial taxa. Finally, while this study explains the causality of several adaptive features (dietary mainly), future work should also investigate the causality of other functional features like disease (virulence) and AMR dissemination, which we only touched upon briefly here.

## Material and methods

### Sample collection

The collection comprised two chimpanzee populations, one indigenous human population (Hadza), adult (non-indigenous) human population from Mahale Mountains near Lake Tanganyika and urban school children from surrounding areas in Moshi Tanzania (Figure 1(a)). The entire selection was a mixture of both biological sexes (male and female), and all the included adults were of various ages except for the school children group (aged 5–14 years old). The selected Hominidae hosts were chosen to represent a wide range of primate hosts from Tanzania to reflect not only various evolutionary history, but also distinguished lifestyles between all five populations (Table S1).

After obtaining permission from village chiefs and village councils, women’s focus groups were formed and trained on proper protocols to assist with gaining subject consent, and ensuring standardized processes and procedures to safely acquire fresh, uncontaminated samples. A total of 116 samples were collected from non-indigenous urban human (adults) residing in and around Mahale Mountains between July and September 2015. From school children, 292 fecal samples were collected from individuals in P1 to P7 school class levels (between 6–15 years old) from 15 different schools in 2015. All schools were geographically close and within Moshi area. Regarding school children samples, after receiving consent from the respective head teachers, who gathered all children’s parent approvals, biosamples were collected from school children in the form of stool specimens. The initial analysis aimed to detect intestinal worms and parasites in these stool samples. This analysis took place approximately one week after the annual deworming program routinely conducted among school children in Tanzania. In instances where children tested positive for stool parasites, their parents were promptly notified and advised to seek further medical evaluation and consultation at KCMC (Kilimanjaro Christian Medical Centre) hospital. From indigenous human population, 48 fecal samples (all adults) were collected from one of the last hunter gatherer populations (Hadza, location Sengeli) in Tanzania near Lake Eyasi in November 2015. Despite the ongoing disruption and alteration of the Hadzabe community, due to several political and ecological changes, that caused the community to shrink and change its traditional lifestyles (e.g., increased exposure to medicine and different dietary items), Hadza is still considered one of the last Indigenous communities to practise their traditional lifestyles more often. For chimpanzees, 27 and 63 fecal samples were collected from two wild chimpanzee populations in Tanzania, Issa Valley (in 2016–2017) and Mahale (in 2015) respectively. Both chimpanzee populations are in the Greater Mahale Ecosystem (GME) area in Western Tanzania. Regarding Mahale chimpanzee samples, human-habituated chimpanzees at the Mahale Mountains National Park^83^ tolerate observation and fresh uncontaminated fecal samples were collected concurrently at the time of human sample collection at Mahale between July and September 2015. All chimpanzee tracking, observations, and sample collection were performed while wearing a facemask (covering nose and mouth) and all activities were done in accordance with Tanzania National Park Guidelines which includes, maintaining a distance of at least 10 m, not eating or drinking in the presence of chimpanzees, and not leaving personal belongings on the ground. All fecal samples were collected fresh immediately after defecation, including the chimpanzee samples. Sample collectors wore a facemask and sterile gloves. Using a fresh, sterile wooden spatula, feces from the top layers (not in contact with soil in the case of chimpanzee collection) were collected. The samples for metagenomics analyses were submerged in RNA later and remained on frozen gel packs if they were collected in remote areas, and remained chilled on frozen packs until a −20°C freezer was available at the local field laboratory. All the samples were then transported on dry ice and stored at KCMC at −80°C until DNA extraction. Samples were transferred by personnel wearing face masks and gloves, in addition to other protective gear. This research was conducted in compliance with the American Society of Primatologists’ Principles for the Ethical Treatment of NonhumanPrimates, and complied with approved protocols and adhered to the legal requirements of Tanzania and the United States.

All population names and wording are carefully chosen to express cultural appropriation as recommended by indigenous community members and members of the Hadza (Hadzabe) community of hunter-gatherers in Tanzania, East Africa (see Mangola et al.^84^ for a perspective on microbiome research ethics in Indigenous communities), (please see section Recognition of work with indigenous and non-indigenous communities in Tanzania below for details).

Additionally, we obtained supplementary ethical approval from NIMR-Tanzania for the study and sampling and to proceed with the metagenomics analysis, and from the Tanzania National Parks (TANAPA), Tanzania Wildlife Research Institute (TAWIRI), and Tanzania Commission for Science and Technology (COSTECH) for all additional sampling (please see section Ethical Declarations below for details). All biosample collections were de-identified and conducted with the required ethical clearance.

Ten samples were also sequenced in triplicates to ensure reproducibility, and all triplicates were identical in their microbiome composition. In the phylogenetic analyses, we selected one representative sample from each triplicate, as this approach avoids redundancy and provides clearer and more accurate insights into evolutionary relationships. We analyzed triplicates separately in the PCA (Figure 3) to assess technical consistency and ensure that observed variations in microbiome composition were due to biological differences rather than sequencing artifacts. We confirmed overlap and high level of similarity between the triplicates (Similar to).^85^

### DNA extraction and library preparation

Total genomic DNA was extracted from all samples with the QIAamp Fast DNA stool mini kit (Qiagen, Hilden, Germany) following the manufacturer’s instructions with the following modifications: 200 mg of feces was kept on ice and used as a starting material and mixed with 1000 µl InhibitEX Buffer. TissueLyser (Qiagen) was used for bead treatment in two cycles of 1 min at 30 hz. A total of 30 μl proteinase K and 400 μl AL Buffer were used for lysing, and 400 μl Ethanol 99% was used for protein precipitation. Finally, the DNA was eluted in two volumes of 50 µl of pre-heated (65°C) AE buffer to increase DNA yield. In addition to two DNA extraction kit controls that were added to each DNA extraction set. All those controls had unmeasurable amounts of DNA using our DNA quantification method (Qubit). Library preparation was attempted on those controls but all their libraries have failed due to the unmeasurable amount of DNA and therefore not included for any further analyses. Genomic DNA quality was assessed using TapeStation Genomic DNA Assay (Agilent Technologies, Santa Clara, CA). Library preparation was performed using the KAPA HyperPrep kit without PCR as per the manufacturer’s recommendations (Kapa Biosystems, Roche, Basel, Switzerland). Library quality and quantity were assessed with the Qubit 2.0 DNA HS Assay (Thermo Fisher Scientific, Waltham, MA) and QuantStudio® 5 (Applied Biosystems, Foster City, CA). Libraries were loaded onto an Illumina Nova-Seq S4 PE 150 cycle format to target 80 M total reads (40 M reads each direction) per sample. However, due to the sequencing process, the actual sequencing depth often exceeded this target, resulting in an average of 107 million reads per sample in our study.

### Read assemblies, taxonomical and gene assignment, and binning

The paired-end reads were quality- and adapter-trimmed using Bbduk2 (version 36.49,^86^ which is part of the Bbmap suite of NGS tools. Trimming removed adaptors, and right-end trim was applied to remove bases with Phred scores below Q20. Lastly, reads shorter than 50 bp were also removed. All the high quality trimmed reads were assigned to bacteria, DNA viruses, bacteriophages, fungi, protozoa, parasites, plants and animals using KMA 1.3.27^87^ to align the reads to the required database: bacterial database (contains complete and partial bacterial genomes from all NCBI accessions by the time of the alignment and were manually curated at DTU (similar to Hendriksen et al.^33^ DNA viruses (contains complete and partial DNA viral genomes from all NCBI accessions by the time of the alignment and were manually curated at DTU), large bacteriophages (adapted from Al-Shayeb et al. 2020,^40^ fungal, protozoa and parasites databases are all partial or complete genomes from NCBI by the time of the alignment). For plant and animal DNA, the database was comprised of all mitochondrial DNA (mtDNA) in NCBI by the time of the alignment. For antimicrobial resistance, ResFinder database (2020–08–28)^54^ was used to assign antimicrobial resistance genes to all our hominid microbiomes using KMA 1.3.27.^87^ To compare our Tanzanian hominid microbiomes to other microbiome data of the similar parameters, 179 fecal samples from adults or teenagers containing more than 20 million reads were selected from Almeida et al.^20^ and mapped to the bacterial database. The hosts in this sample set were from Asia (*n* = 72), Europe (*n* = 92), or North America (*n* = 15).

All our high quality trimmed reads were also de novo assembled using metaSPAdes (SPAdes v.3.13.0).^88,89^ N50 values were obtained for all assembled samples using bbmap stats.sh (BBtools may be cited using the primary website: BBMap – Bushnell B. – sourceforge.net/projects/bbmap/). The number of reads were found from the QC summary report from BbMap and BBtools. The average contig lengths and number of contigs from each sample were obtained from the assembly-output files from metaspades. All gene annotation was carried out using Prodigal (v. 2.6).^90^

### Genome binning

Genome binning, quality checking, and taxonomical annotation were done using VAMB(v3.0.1),^36^ CheckM (v1.1.3),^34^ and GTDB-Tk (Release 220, April 2024)^38^ with the release 89 database, respectively, and similar to Jespersen et al.^91^ All Near Complete (NC) MAGs were de-replicated to unique MAG species with 95% Average Nucleotide Identity (ANI), using dRep compare (v2.2.3).^92^ The best representative MAG was selected for each MAG species as the one with the best score from the dereplication. Reads from all our Hominidae Tanzanian microbiomes and the 179 samples from Almeida et al.^20^ were mapped with CoverM (v0.6.1) (Woodcroft, B. CoverM. https://github.com/wwood/CoverM.) to the catalog of MAG species (best representatives) from this study and the MAG species identified from the Almeida samples in Nissen et al.^20,36^ For the core genome, differential abundance, and ordination analyses, only the reads from the Tanzanian microbiomes from this study were mapped to the unique MAGs species identified from the same samples. The read counts were rarefied to 1,014,729 (minimum number of counts in a sample) and filtered based on a coverage ratio threshold (expected/covered ratio of a genome ≥ 0.5) prior to calculating abundances as Transcripts per Kilobase Million (TPM).

### Phylogeny and functional annotation

Phylogenetic trees containing all NC MAGs were created using FastTree (v2.1.11)^93^ based on the marker gene set from GTDB-Tk. Phylogenetic analyses of single MAG species and functional annotations of these were done following the same procedure as in Jespersen et al.^91^ Additionally, we also inferred species phylogenies with CSI phylogeny^94^ using MAG fasta genomes as input and the best representative genome for each MAG species as a reference. In these trees, multiple genomes identified as the same species from another study were also included.^36^ The trees created with CSI phylogeny were based in 15.53% (C4), 27.63% (C7), and 9.78% (C15) (which were comparable to previous studies^91^ of the positions in the best representative genome.

### Richness, alpha diversity and ordination (beta diversity) analysis

Richness and alpha diversity were both calculated based on the rarefied, TPM abundances. Richness was identified as the number of observed species (either unique MAGs or from database mapping with KMA) and Shannon diversity index was calculated using the Vegan package in R (Vegan^95^ R package version 2.5–6. https://CRAN.R-project.org/package=vegan).

The ordination analysis was done according to standard practice, by first centering the data around the geometric mean and scaling the data to unit variance and then by calculating eigenvectors and -values of the centered log-ratio (CLR)-transformed counts using SV-decomposition. Before the data were CLR-transformed, the zero valued features were replaced using Bayesian point estimation. Two principal components were selected and data were projected onto the plane spanned by them. The resulting biplots were visualized by python (3.11.4) and Matplotlib (3.7.1). Genomic features were shown as labeled arrows while samples were represented by colored density contours. The contours were truncated at 10% of the peak value and the remaining samples below this level were shown as individual points in the same color as the corresponding contoured regions. The proportion of explained variance per component was shown in parenthesis in the axis label.

In some cases, a certain amount of feature reduction was required for the PCA to produce meaningful output. First of all, PCA is only valid when the number of features is smaller or equal to the number of samples. Secondly, because the biplot shows variance in the data, a large number of low-variance features will only smear out and obfuscate any signal present in the data. Additionally, low-abundance features will be dominated by Poisson noise in count space, potentially leading to high noise-dominated variance in CLR-space. Therefore, features with low variance *or* low mean CLR abundance were filtered out, effectively reducing the dimensionality of the feature space. A maximum of 100 features per PCA was arbitrarily chosen as a good trade-off between filtering noisy or low-variance features out while keeping important features in.

### Core bacterial community analyses and differential abundance analysis and statistical analyses

The core bacterial communities in the hominid populations and between the hominid groups were all calculated similarly to Otani et al.^96^ for both read mapped output and MAG taxonomy.

Differential abundance analysis was used to determine taxonomic differences in the data composition. Again, the reads mapped to different databases (unique MAGs or reference database) were used. Differential abundance analysis was also used to determine the variations in antimicrobial resistance genes in all the included microbiomes.

The differential abundance analysis was made with the R-package ALDEx2 version 1.18.0^97^ and the data was grouped and compared in pairs of two at a time: *Homo vs Pan* and indigenous human community (Hadza) *vs n*on-indigenous humans (children and adults).

Differences between these groups in regards to their centered-log ratio (CLR) abundance was tested by a Welch’s t-test and corrected by a Benjamini-Hochberg false-discovery rate (FDR) correction. The effect size was used to quantify how different two distributions were from each other.

The measure effect was returned with this analysis and was used to quantify significant drivers between groups. Effect is calculated as the median difference of the CLR values between two groups (diff.btw) divided by the median of the largest CLR difference within groups (max(diff.win)), following the equation: effect = (diff.btw)/(max(diff.win)).

The top 10 most statistically significant features driving each group were reported in the main text.

### Functional analysis

We also investigated the compositional functional differences between groups.

EggNOG version 2.1.6^98^ was used to do the functional annotation on the Prodigal predicted proteins of the samples. Output files from EggNOG were combined into one file per host population group.

Functional differences between groups were investigated using the results from the KEGG column in the EggNOG output. Some of the protein entries had more than one k-number, suggesting the protein was associated with more than one function. This makes biological sense, and the multiple k-numbers were added with count-value 1. This means that one protein could contribute with more than one k-number.

### KEGG analysis

The KEGG Orthology (ko)^53^ was downloaded (2. May 2022) and a Python script was written to parse the KEGG Orthology results from the EggNOG analysis and trace each of the K-numbers to their detailed functional annotations. Then the files were imported into R for visualization.

### CAZyme annotation and functional prediction

Proteins predicted by Prodigal from the 576 metagenomes were identified using the rapid k-mer clustering program CUPP.^99^ The algorithm employs a nearest-neighbor approach, dividing CAZy families into small clusters and assigning a function only if at least one characterized member is found in the same cluster.

### Substrate specificity

General substrate groups were assigned to the functionally predicted sequences based on a thorough inspection of the BRENDA database^100^ and a review of literature in CAZy^101^ and CAZypedia^102^ for each family-EC combination. In cases of ambiguity, the substrate group most relevant and likely for gut bacterial CAZymes was chosen. The substrate categories were further classified into three major sources for a convenient overview: Plant-glycans (covering plant and algae glycans), Host-glycans (encompassing glycoproteins, proteoglycans, cerebrosides, lactose, etc.), and Microbial-glycans (including bacterial and fungal polysaccharides).

For plotting relative abundances of the CAZyme output, count of genes targeting a specific function in a host population were divided by the total count of CAZyme genes that were found within that host population. This was done for all CAZyme genes identified within all the MAGs (Figure 4(c)), and for the CAZyme genes identified within all MAGs within a genus of interest (Figure 5(b,d); Figure 6(d-g); Figure S4).

### Statistical analysis of relative abundance data

#### Differential abundance analysis of taxa:

Differential abundance analysis was used to determine taxonomic differences in the data composition between host groups. The differential abundance analysis was made with the R-package ALDEx2.

The function aldex() was used to do the differential abundance analysis. First, this function performed a centered log-ratio CLR transformation on the data using the aldex.clr module. Then, the aldex.ttest module performed a Welch’s t-test and a Wilcoxon rank test. Lastly, the function estimated the effect size with the aldex.effect module. The effect size was used to quantify how different two distributions were from each other i.e. how many standard deviations their mean is apart.

The measure effect was returned with this analysis and was used to quantify significant drivers between groups. Effect is calculated as the median difference of the CLR values between two groups (diff.btw) divided by the median of the largest CLR difference between two groups (max(diff.btw)).

An absolute threshold of 1 was used to define significantly different taxa between groups as suggested by the documentation and examples provided by ALDEx2.

#### Testing of richness, diversity and taxonomy differences:

Population differences in diversity or richness were tested with a pairwise Wilcoxon test. P-values were corrected for multiple testing, using the BH algorithm. Taxonomic differences phylum distributions were tested with pairwise Kolmogorov–Smirnov tests and adjusted for multiple testing as well, (full list of the test output are in Tables S2, S3).

#### Cazyme functional analysis:

The relative abundance data for each substrate, grouped by human and non-human hosts, were statistically analyzed to assess differences in abundance. Due to the compositional nature of the data, a centered log-ratio (CLR) transformation was applied. Prior to transformation, zeros were replaced with a pseudocount of 1 × 10^−6^ to enable log-transformation. The CLR transformation was computed as:

CLR(x_i) = log(x_i/g(x))

where x_i is the relative abundance of a given substrate, and g(x) is the geometric mean of all relative abundances for that substrate across hosts.

To compare CLR-transformed values between human and non-human hosts for each substrate, a bootstrap resampling approach was employed. This non-parametric method generated 10,000 bootstrap replicates of the observed differences between the two groups (human and non-human) for each substrate. From the bootstrap distribution, 95% confidence intervals (CIs) were calculated. A difference was considered statistically significant if the CI excluded zero (full list of the test output are in Table S7).

All statistical analyses on the Cazyme were conducted using Python, libraries NumPy and SciPy for numerical computations and resampling.

### Virulence gene analyses

To identify virulence gene factors in our MAGs, we first downloaded the latest version of the nucleotide and protein sequence dataset of the VFdb database (downloaded on July 1st 2023.^103^ Then, using the non-dereplicated dataset of all 15,754 MAGs, for each MAG we first identified the predicted amino acid sequences using prodigal^90^ and then used diamond blast^104^ to align them against the VFdb protein dataset using an e-value cutoff of 1e-15, a minimum percentage identify of 50% and a minimum coverage length of 70%.

### Ethics declarations

#### Ethics approval and consent to participate

All human fecal samples were noninvasive (after normal defecation) and anonymized and de-identified, and extra precaution was taken by eliminating all human DNA before submitting it to NCBI even though it is anonymized. No animal experiments were carried out in this project.

The samples were collected legally and ethically with consent and documented approval from the Government of Tanzania and in consultation with field-guides and village councils in Tanzania, permission for the study and sampling was obtained from the National Institute of Medical Research (MR/53i 100/83, NIMR/HQ/R.8a/Vol.IX/1542, NIMR/HQ/R.8c/Vol.II/322 and NIMR/HQ/R.8c/Vol.II/616) and the Tanzania Commission for Science and Technology. Additionally from Mahale specifically, permissions and ethical oversight were granted not only by NIMR but also by Tanzania National Parks (TANAPA), Tanzania Wildlife Research Institute (TAWIRI), and Tanzania Commission for Science and Technology (COSTECH): Permit No. 2014-367-ER-2012-107. A material transfer agreement with the National Institute for Medical Research in Tanzania specifies that the stool samples collected are used solely for academic purposes. All the samples were anonymized upon arrival at DTU and we would not be able to trace the sample back to a host.

## Supplementary Material

Supplemental Material

## Data Availability

The raw sequencing data (FASTQ) generated in this study have been deposited in the European Nucleotide Archive and can be accessed without restrictions under Project ID PRJ EB58441 and Study ID: ERP143500. They also contain the assemblies of all the MAGs that have been produced within this study under the same Project and Study ID. Table S1 contains the sample, experiment and run accessions. Source data are provided with this paper. This study also utilized the publicly available databases of ResFinder, PLSDB, Silva and Kraken 2.

## Inclusion and diversity

We support inclusive, diverse, and equitable conduct of research.

## Recognition of work with indigenous and non-indigenous communities in Tanzania

Research involving indigenous communities is needed for a variety of reasons including to ensure that scientific discoveries and understanding appropriately represent all populations and do not only benefit those living in European or North American nations (For example Abdill et al., 2022.^105^ Special considerations must be made to ensure that this research is conducted ethically and in a non-exploitative manner. In this study, we performed deep metagenomic sequencing on anonymized fecal samples that were collected from the indigenous population Hadza hunter-gatherers in 2013/2014, from local villagers residing in chimpanzee habitat and chimpanzees at Mahale Mountains in 2015 and school children in Moshi in 2015/2016. The samples were collected legally and ethically with consent from the Government of Tanzania and in consultation with field-guides and village councils in Tanzania.
