# Supplementary material for: Genomic and functional co-diversification imprint African Hominidae microbiomes to signal dietary and lifestyle adaptations
Source: Gut Microbes. 2025 Mar 31;17(1):2484385. doi: 10.1080/19490976.2025.2484385 (PMC11959905; doi:10.1080/19490976.2025.2484385)
Supplement: Supplemental Material [file KGMI_A_2484385_SM6521.docx]

**Supplementary Information for**

Genomic and functional co-diversification imprint African Hominidae microbiomes to signal dietary and lifestyle adaptations

Saria Otani^1*#^, Marie Louise Jespersen^1,2*^, Christian Brinch^1^, Frederik Duus Møller^1^, Bo Pilgaard^3^, Emilie Egholm Bruun Jensen^1^, Pimlapas Leekitcharoenphon^1^, Christina Aaby Svendsen^1^, Amalie H. Aarestrup^1^, Tolbert Sonda^4,5,6^, Teresa J. Sylvina^7,8^, Jeff Leach^9^, Alexander Piel^10,11^, Fiona Stewart^10,11,12^, Panagiotis Sapountzis^13^, Paul E. Kazyoba^14^, Happiness Kumburu^4^, Frank M. Aarestrup^1^

^1^ Research group for Genomic Epidemiology, National Food Institute, Technical University of Denmark

^2^ Novo Nordisk Foundation Center for Protein Research, Faculty of Health and Medical Sciences, University of Copenhagen, Copenhagen, Denmark.

^3^ Department of Biotechnology and Biomedicine, Section for Protein Chemistry and Enzyme Technology, Technical University of Denmark

^4^ Biotechnology Research Laboratory, Kilimanjaro Clinical Research Institute (KCRI), Moshi 25102, Tanzania

^5^ Kilimanjaro Christian Medical Centre (KCMC), Tanzania

^6^ Kilimanjaro Christian Medical University College (KCMUCo), 2240 Moshi Kilimanjaro, Tanzania

^7^ National Academies of Sciences, Engineering and Medicine, Washington, DC, USA

^8^ Department of Veterinary and Biomedical Sciences, The Pennsylvania State University, PA, USA
^9^ Microbiome Network and Department of Agricultural Biology, Colorado State University, Fort Collins, CO, USA.

^10^ Department of Human Origins, Max Planck Institute of Evolutionary Anthropology, Leipzig, Germany

^11^ Department of Anthropology, University College London, London, UK

^12^ School of Biological and Environmental Sciences, Liverpool John Moores University, Liverpool, UK

^13^ INRAE-UCA, UMR 0454 MEDIS, Clermont-Ferrand, France

^14^ National Institute for Medical Research, 3 Barack Obama Drive, Dar-Es-Salaam, Tanzania

* Share first co-authorship

^#^ Corresponding author: [saot@food.dtu.dk](mailto:saot@food.dtu.dk)

**Supplementary Tables:**

(Please NOTE: All below tables are too large to be uploaded into the submission portal, please find them on this link instead: <https://drive.google.com/drive/folders/1ds-0_xvmeCbO_AmZJnAhK_UHRX9xIGTz?usp=sharing> )

Tabel S1: An overview of all the included Hominidae gut microbiomes, with all their known metadata including population source and location, sequence ID to match the ENA accession number. Sheets 2 and 3 contain the ENA accession numbers for both the microbiome samples and their assemblies. Sheet 4 contains detailed base-pair number and read depth per sample.

Table S2: All bacterial species identified in all hominid guts when mapping the high-quality reads (not assemblies) to our custom-made bacterial database (sheet 1 separate for each sample - sheet 2 grouped into populations). Third sheet shows statistical variations in diversity indices (richness and diversity) calculated using a pairwise Wilcoxon test. P-values were corrected for multiple testing, using the BH algorithm.

Table S3: All the unique MAGS (metagenome-assembled genomes) identified in our hominid gut microbiomes. With their abundances (TPM) based on the reads mapped back to those MAGs used as an alignment database. First sheet contains the abundances of the reads mapped to MAGs from this study only. Second sheet contains the abundances of these reads/MAGs combined with the reads and MAGs from Nissen *et al.*, 2021 and Almeida *et al.*, 2019 (1, 2). Third sheet contains taxonomical annotations of all NC MAGs. Fourth sheet shows statistical variations in diversity indices (richness and diversity) calculated using a pairwise Wilcoxon test. P-values were corrected for multiple testing, using the BH algorithm.

Table S4: All non-bacterial gut components from the Hominidae samples. Each sheet contains a group of organism DNA. Those are the inputs for Figure 4A and 4B, and Figure S1.

Table S5: All antimicrobial resistance genes (read counts) and classes (CLR values) in the Tanzanian Hominid microbiomes. In addition to the results of the differentially abundant analysis conducted on those resistomes.

Table S6: CAZyme analysis output. With contig labels, gene annotation and counts, bacterial assignment and predicted substrate and suggested plant, animal or microbial glycan. This table contains all CAZyme hits that belonged to bacterial contigs with and without bacterial taxa assignments.

Table S7: Sheet 1: CAZyme analysis output. With contig labels, gene annotation and counts, bacterial assignment and predicted substrate and suggested plant, animal or microbial glycan. This table contains all CAZyme hits that belonged to bacterial contigs only with bacterial taxa assignments. All following sheets show statistical analysis outputs of the Cazymes in individual bacteria MAGs. All values are CLR-transformed for each substrate, and a bootstrap resampling approach was employed to generate 10,000 bootstrap replicates of the observed differences between the two groups (human and non-human) for each substrate. From the bootstrap distribution, 95% confidence intervals (CIs) were calculated. A difference was considered statistically significant if the CI excluded zero. This was done on all data presented in Figures 5 and 6 and all the ones in Figure S4.

Table S8: Core bacterial community that is shared between the five hominid populations. Based on the high quality read mapped to our custom-made bacterial database (based on Table S2).

Table S9: Core bacterial community that is shared between the five hominid populations. Based on all the unique MAGS (metagenome-assembled genomes) identified in our hominid gut microbiomes (based on Table S3).

Table S10: Taxonomical annotations and PERMANOVA statistics of 14 unique MAGs that were present in more than 100 microbiome samples, in addition to C15 MAG (*Brachyspira*) that was added due to its increased abundance in the Hadza microbiomes.

Table S11: All KEGG pathways that were detected in our Hominidae microbiomes.

Table S12: All identified virulence genes, within their MAGs assigned to bacterial taxa, in all our Hominidae microbiomes.

**Supplementary Figures**

(Please NOTE: All below figures are too large to be uploaded into the submission portal, please find them on this link instead: <https://drive.google.com/drive/folders/1Xe6CRgq_NRrE_WgQnNvBKqzEvaA_wBIs?usp=sharing>)

Figure S1: Ordinations of all microbiome members: bacteria (at various taxonomical levels), DNA viruses, large phages, mitochondrial DNA, fungi, parasites, ARGs (antimicrobial resistance genes), plastid and protozoa. All based on principal component analysis (PCA) visualising that all hominid microbiomes from all five populations are influenced by the hosts. The contours show the microbiome density in each population (each colour) and are truncated at 50% of the peak value. Microbiomes outside this range are represented as individual points with the same colouring scheme. All principal components in the panels are calculated from centred log-ratio (CLR)-transformed size-adjusted counts. Bar charts of the mtDNA read counts for each of plant, farming animal and bees in all five hominid groups included.

Figure S2: Pathway annotation based on KEGG Orthology analysis within the Organismal Systems category between the five hominid populations. The fractions of each KEGG pathway represent the relative abundance of the genes involved in the assigned function.

Figure S3: Top 20 statistically significant bacterial MAGs between human and chimpanzee gut microbiomes, and between Hadza and non-indigenous human microbiomes. With their taxa assignment and MAG numbers. Statistically significant bacterial genera are identified with differential abundance analysis on centred log-ratio (CLR)-transformed size-adjusted counts and adjusted with Benjamini-Hochberg (BH) and false-discovery rate (FDR) correction <0.05 (see **Table S3** for MAG numbers and full list).

Figure S4: Relative abundances of CAZyme-encoding genes targeting different dietary substrates in *Faecalibacterium*, *Alistipes*, *Dorea*, *Brachyspira*, UBA11524, *Prevotella*, *Mogibacterium* and *Blautia* MAGs, showing differences in functional annotations of those CAZymes between *Homo* and *Pan* or between non-indigenous adults and children, calculated per sample as the gene counts encoding a CAZyme function and then corrected by the total CAZYme gene counts per population based on CAZyme analyses (see Methods for details). Each substrate is shown as two bars/boxes with different colours representing either all human or all chimpanzee microbiomes, or all non-indigenous adults or all non-indigenous children microbiomes. In bar plots sample relative abundances are summed per population. In box plots relative abundances are shown per sample.

Figure S5: Barplots of *Faecalibacterium*, *Alistipes*, *Dorea*, UBA11524, *Prevotella*, *Mogibacterium* and *Blautia* MAGs that were identified in all our hominid microbiomes. Top plot contains all MAGs assigned to each taxon. Below those are all barplots of all differentially abundant bacterial MAGs that we identified between the different Hominidae groups that are in Figure 3D,3F and Figure S3. Abundances are calculated as transcripts per kilobase million (TPM) per sample and coloured according to population.

Figure S6: Strain-level phylogenetic analysis of the selected MAGs in Table S10, all visualised by ASTRAL tree. Population R^2^ values from PERMANOVA testing of these trees are present in Table S10. The tree tips are coloured according to their hominid population.

Figure S7: Comparison between a number of our bacterial MAGs and the same bacterial taxon MAGs/genomes (ANI > 95%) from other studies (1-3) using strain-level phylogenetic analysis visualised by SNP trees. The taxa included are UBA11524, *Methanobrevibacter* with two varying external MAGs (not only from this study) and C15 *Brachyspira*. The tree tips are coloured according to their hominid population (our MAGs) or WHO region (other data).

Figure S8: Host Population R^2^ values of C4, Christensenellales UBA11524 split by gene groups (GO annotation). A: Host population R^2^ values of gene trees divided by their biological process group. Groups with less than 5 genes in them were combined with the group of unknown annotation (NA). B: Separation of the biological process group metabolic process (blue in plot A) into sub annotation groups. Metabolic process groups with less than five genes in them were excluded from the plot. Gene trees with population R2 values above 0.5 were investigated further (Figure 6C). C: SNP trees (maximum likelihood) of single genes from Christensenellales UBA11524 MAGs of three genes with high population R2.

Figure S9: Presence of virulence genes in common pathogenic bacteria members in Escherichia, Klebsiella, Enterobacter, Pseudomonas, Haemophilus, and Citrobacter MAGs across all our Tanzanian microbiomes. All virulence genes are represented by their virulence factor category (left side), and the relative abundance of those genes in each MAG are represented in log-transformed scale within the heatmap and based on our tailored zoonotic VFdb; refer to "STAR Methods".

**Supplementary Results:**

Bacterial taxa that were significantly abundant in a hominid population over another:
*Oscillibacter* (*e.g.,* MAG 2464), *Blautia* (*e.g.,* MAG 4474), various genomes of *Faecalibacterium* and *Dorea* (*e.g.,* MAG 2784), two *Gemmiger* genomes, and *Agathobacter* (MAG 62058) were significantly more abundant in human guts than in chimpanzees (**Figure 3D, Figure S3**). Lachnospiraceae UBA7160, *Blautia* (*e.g.,* MAG 1851), *Dialister* (*e.g.,* MAG 4707), *Eubacterium* (*e.g.,* MAG 2014), *Solobacterium* (*e.g.,* MAG 8639), *Dorea* (*e.g.,* MAG 2822), *Senegalimassilia* (*e.g.,* MAG 3990), *Collinsella* (*Enorma - e.g.,* MAG 2928), two *Parasporobacterium* genomes, and *Mogibacterium* (*e.g.,* MAG 43 & 1170, the latter only in chimpanzee microbiomes) were amongst the most significantly abundant bacteria in chimpanzees (**Figure 3D, Figure S3**). For humans, Hadza microbiomes harboured a distinct subset of bacterial taxa: *Collinsella* (*Enorma - e.g.,* MAG 17269), *Slackia* (MAG 5484), *Campylobacter* (*e.g.,* MAG 1018), *Olsenella* (*e.g.,* MAG 8257), *Solobacterium* (*e.g.,* MAG 3268), *Brachyspira* (*e.g.,* MAG 619), and *Mogibacterium* (*e.g.,* MAG 129) (**Figure 3E, Figure S3**). Two *Agathobacter* genomes, two *Faecalibacterium* genomes, *Dorea* (e.g., MAG 2640), *Alistipes* (*e.g.,* MAG 97), *Holdemanella* (*e.g.,* MAG 2591), *Roseburia* (*e.g.,* MAG 712), and *Eubacterium* were abundant in non-indigenous human populations (adults and children) (**Figure 3E, Figure S3**).

Functionally different bacterial taxa:
The two *Dorea* MAGs (NODE2822 and NODE 2784) had separate functions in human and chimpanzee gut microbiomes (**Figure S4; Table S7**). In non-indigenous humans, *Dorea* MAGs encoded high levels of β-fructofuranosidase, which targets fructans in fruit (**Figure S4; Table S7**). In chimpanzee guts, *Dorea* MAGs encoded several enzymes with capacity to degrade plant substrates through α-glucosidase to degrade α-1,4-glucan in plant starch, in addition to animal-glycan degrading enzymes such as α-N-acetylglucosaminidase (**Figure S4; Table S7**).

*Faecalibacterium*, which was significantly more abundant in human guts (**Figure 3D**), had very distinct functions in humans and chimpanzees (**Figure S4, Table S7**), being involved in more animal substrate degradation (e.g., glycoproteins and lactose) in human microbiomes (**Figure S4; Table S7**). It is also involved in 1,3-α-L-fucosidase production, which cleaves 1,3-linkages of fucose residues in human milk oligosaccharides (HMOs) (4). More specifically, in children, *Faecalibacterium* increases lacto-N-biosidase to break down milk oligosaccharides (lacto-N-tetraose) into disaccharides and simple sugars lacto-N-biose I and lactose, which finally produce GDP for energy (5 - **Table S7**). *Faecalibacterium* was entirely involved in plant-based diet breakdown in chimpanzee microbiomes (**Figure S4, Table S7**), producing higher levels of plant degrading enzymes endo-1,4-[β](https://biocyc.org/gene?orgid=META&id=MONOMER-16269)-D-glucanase, endoglucanase G, and α-N-arabinofuranosidase for cellulose and hemicellulose degradation (**Table S7**).

Within human microbiomes, there were further minor functional differences between groups. *Alistipes* MAGs were more abundant in non-indigenous microbiomes (**Figure 3E, Figure S3, Figure S5**), with CAZyme analyses assigning functions only to its non-indigenous hosts (**Figure S4, Table S7**) (with the exception of one CAZyme encoding gene from single Hadza sample (not shown in plot)). It encoded enzymes involved in degrading both plant and animal substrates: in non-indigenous adults, it encoded higher levels of endo-β-1,4-galactanase to degrade plant pectins and β-N-acetylhexosaminidase to break down animal tissues (glycoprotein) (**Figure S4, Table S7**), while in children it encoded enzymes that target fruits (fructan β-(2,1)-fructosidase) and beta-glucan in plants ( β-glucosidase and a large subset of plant-degrading enzymes) (**Figure S4, Table S7**).

Neurological processes in Hominidae:
Neurological processes were also impacted by variations in human and chimpanzee gut microbiomes, with chimpanzee microbiomes more involved in glutamate signalling, an essential excitatory neurotransmitter pathway tightly linked to cognitive demands reflecting social interactions, problem-solving, and spatial navigation in the wild. Chimpanzee microbiomes also harboured bacteria with opioid (morphine) degradation pathways (**Figure 7B**), suggesting the consumption of opioidergic plants in their habitats. Conversely, in indigenous and non-indigenous humans, microbiomes were more involved in endocannabinoid signalling (pain, mood, and appetite sensors) (**Figure 7B**). Specifically, Hadza microbiomes were enriched for bacteria involved in neurotransmitter interactions (like dopamine and serotonin) and alcohol consumption and addiction (**Figure 7B**), consistent with previous observations of opioidergic plant consumption in the Hadza (6 and personal observations).

Comparing Tanzanian and external strains:

To compare the Tanzanian strains to global strain variation, we wanted to include strains from other countries in our phylogenetic trees of four interesting species. Of these four, two were selected based on their high population R2 values (C4: UBA11524 and C7: *Methanobrevibacter*), and two others based on functional variations (C10: *Prevotella* and C15: *Brachyspira*). To assess global strain variation we downloaded all genomes of these four genera from two previous publications (Nissen et al. 2021 and Pasolli et al. 2019 (1, 3)). For *Prevotella*, we downloaded 5,111 genomes, however, none of these were within our ANI threshold (>95%), and we could not include these in our phylogenetic species tree, suggesting that the global variation of *Prevotella* spp. is more than 5% ANI, due to a high genome diversity within this genus. Another explanation for the difficulties in identifying *Prevotella* genomes similar to ours could be due to the linkage between *Prevotella* and a non-western lifestyle and the sampling bias against samples from such lifestyles. For the other species, we were able to find some genomes of high enough similarity to be included in our phylogenetic trees, in the case of *Methanobrevibacter*, some of the identified genomes were more different than others, leading to a clear separation in the tree (**Figure S6**). If we removed these three genomes from the tree, we were able to see the six remaining genomes still separated from the Tanzanian strains in the tree.

**Supplementary Information References:**

1- Nissen, J.N., Johansen, J., Allesøe, R.L. *et al.* Improved metagenome binning and assembly using deep variational autoencoders. *Nat Biotechnol* **39**, 555–560 (2021). https://doi.org/10.1038/s41587-020-00777-4

2- Almeida, A., Mitchell, A.L., Boland, M. *et al.* A new genomic blueprint of the human gut microbiota. *Nature* **568**, 499–504 (2019). https://doi.org/10.1038/s41586-019-0965-1

3- Pasolli, E., Asnicar, F., Manara, S., Zolfo, M., Karcher, N., Armanini, F., Beghini, F., Manghi, P., Tett, A., Ghensi, P., Collado, M. C., Rice, B. L., DuLong, C., Morgan, X. C., Golden, C. D., Quince, C., Huttenhower, C., & Segata, N. (2019). Extensive Unexplored Human Microbiome Diversity Revealed by Over 150,000 Genomes from Metagenomes Spanning Age, Geography, and Lifestyle. *Cell*, 176(3), 649-662.e20. [https://doi.org/10.1016/j.cell.2019.01.001](https://www.sciencedirect.com/science/article/pii/S0092867419300017)

4- Ogata-Arakawa, M., Muramatsu, T., & Kobata, A. (1977). α-l-Fucosidases from almond emulsin: Characterization of the two enzymes with different specificities. Archives of Biochemistry and Biophysics, 181(1), 353-358.<https://doi.org/10.1016/0003-9861(77)90514-8>

5- Sano, M., Hayakawa, K., & Kato, I. (1992). An enzyme releasing lacto-N-biose from oligosaccharides. Proceedings of the National Academy of Sciences, 89(18), 8512–8516. [https://doi.org/10.1073/pnas.89.18.8512](https://www.pnas.org/doi/abs/10.1073/pnas.89.18.8512)

6- Roulette, C.J., Kazanji, M., Breurec, S. and Hagen, E.H. (2016), High prevalence of cannabis use among Aka foragers of the Congo Basin and its possible relationship to helminthiasis. Am. J. Hum. Biol., 28: 5-15. <https://doi.org/10.1002/ajhb.22740>
